# Tetradecyl 2,3-Dihydroxybenzoate Improves Cognitive Function in AD Mice by Modulating Autophagy and Inflammation Through IPA and Hsc70 Targeting

**DOI:** 10.3390/ijms252111719

**Published:** 2024-10-31

**Authors:** Opeyemi B. Fasina, Lanjie Li, Danni Chen, Meijuan Yi, Lan Xiang, Jianhua Qi

**Affiliations:** College of Pharmaceutical Science, Zhejiang University, 866 Yu Hang Road, Hangzhou 310058, China; fasinaopeyemi@zju.edu.cn (O.B.F.); lilanjie@zju.edu.cn (L.L.); 12019025@zju.edu.cn (D.C.); 22119153@zju.edu.cn (M.Y.)

**Keywords:** Alzheimer’s disease, tetradecyl 2,3-dihydroxybenzoate, indole-3-propionic acid, neurogenesis, neuron inflammation, gut microbiota, metabolites, chaperone-mediated autophagy

## Abstract

Drug development for Alzheimer’s disease (AD) treatment is challenging due to its complex pathogenesis. Tetradecyl 2,3-dihydroxybenzoate (ABG-001), a leading compound identified in our prior research, has shown promising NGF-mimicking activity and anti-aging properties. In the present study, both high-fat diet (HFD)-induced AD mice and naturally aging AD mice were used to evaluate anti-AD effects. Meanwhile, RNA-sequences, Western blotting, immunofluorescence staining, enzyme-linked immunosorbent assay (ELISA), cellular thermal shift assay (CETSA), drug affinity-responsive target stability (DARTS) assay, construction of expression plasmid and protein purification, surface plasmon resonance (SPR) analysis, and 16S rRNA sequence analysis were used to identify the target protein of ABG-001 and clarify the mechanism of action for this molecule. ABG-001 effectively mitigates the memory dysfunction in both HFD-induced AD mice and naturally aging AD mice. The therapeutic effect of ABG-001 is attributed to its ability to promote neurogenesis, activate chaperone-mediated autophagy (CMA), and reduce neuronal inflammation. Additionally, ABG-001 positively influenced the gut microbiota, enhancing the production of indole-3-propionic acid (IPA), which is capable of crossing the blood–brain barrier (BBB) and contributes to neuronal regeneration. Furthermore, our research revealed that IPA, linked to the anti-AD properties of ABG-001, targets the heat shock cognate 70 kDa protein (Hsc70) and regulates the Hsc70/PKM2/HK2/LC3 and FOXO3a/SIRT1 signaling pathways. ABG-001 improves the memory dysfunction of AD mice by modulating autophagy and inflammation through IPA and Hsc70 targeting. These findings offer a novel approach for treating neurodegenerative diseases, focusing on the modification of the gut microbiota and metabolites coupled with anti-aging strategies.

## 1. Introduction

Alzheimer’s disease (AD) is widely recognized as a significant contributor to morbidity and reduced quality of life in the aging population. This disease not only places a considerable burden on healthcare resources but also hinders economic development. AD impacts cognitive function, but the exact cause of this phenomenon is unclear. Nevertheless, multiple markers and causes have been associated with this disease. The most widely accepted pathogenic factors include amyloid beta (Aβ), Tau, and neurofibrillary tangles (NFTs); inflammation; synapse loss; disruption of the BBB; the gut microbiome; oxidative stress; and epigenetic modifications [1]. Various drugs, including four cholinesterase inhibitors, one N-methyl-D-aspartate (NMDA) receptor antagonist, one monoclonal antibody aducanumab, and GV-971 [2,3,4], have been used for the treatment of AD. Although these drugs have been shown to effectively manage AD symptoms, it is important to note that they do not offer a definitive cure for this disease [5]. Therefore, it is crucial to actively pursue the identification of new therapeutic targets and strategies and develop novel drugs to combat and prevent AD.

The gastrointestinal tract harbors a complex network of microorganisms consisting of both beneficial and harmful bacteria. This network plays a critical role in the overall health of the host and has a strong connection with the brain [6]. The microbiota has long been recognized to have a significant impact on the development of the nervous system by influencing neurogenesis, microglial maturation, the maintenance of the BBB, and neuronal function. Recent studies have revealed the crucial role of intestinal dysbiosis and gut diseases in AD [6]. Furthermore, research has provided evidence linking an imbalance in the intestinal flora to the pathophysiology of AD. Metabolites produced by the intestinal microbiota, including lipopolysaccharide (LPS) and short-chain fatty acids (SCFAs), such as indole-3-propionic acid (IPA), tryptamine, butyrate, and valproic acid (VPA), have been identified as potential contributors to the prognosis of AD [7]. These components are believed to mediate the gut–brain axis in the context of AD. As a result, there is growing interest in exploring the modulation of the intestinal microbiota as a potential treatment approach for AD in recent years.

ABG-001 is a leading compound that exhibits novel NGF mimetic activity. It was discovered in our previous study and is a synthesized derivative resulting from structure–activity relationship studies. The compound is based on the chemical structure of gentisides found in *Gentiana rigescens* Franch, a Chinese herbal medicine [8]. ABG-001 has been shown to induce neurite growth in PC12 cells by regulating the IGF-1 signaling pathway [9]. In vitro studies have identified the Hsc70 protein as a potential target of ABG-001 [9]. Furthermore, ABG-001 has been demonstrated to ameliorate cognitive dysfunction and white matter damage after cerebral hypoperfusion, inhibit glial cell promotion, mitigate neurodegeneration, and induce recovery of hind limb motor function in patients with spinal cord injury. It also promoted neurite growth and the survival of neuronal cells in Aβ25-35 mice [10,11,12].

Due to the structural properties of ABG-001, its solubility and permeability are lower, resulting in a bioavailability of only 8%. It cannot pass through the BBB and is undetectable in brain tissue during pharmacokinetic and metabolic studies. This finding raises the possibility that ABG-001 may exert its anti-AD effect through alternative mechanisms. Additionally, the accumulation of this molecule in the intestine of rats after oral administration suggests that it may produce anti-AD effects at lower concentrations via the gut–brain axis.

The Hsc70 protein is a chaperone that plays a crucial role in chaperone-mediated autophagy (CMA), a type of autophagy that is linked to metabolism, DNA repair, and T-cell activation [13,14]. It has the ability to selectively clear substrates that contain the Lys-Phe-Glu-Arg-Gln (KFERQ) motif. CMA recruits substrate proteins to lysosomes through heat shock protein family A (Hsp70) member 8 (Hsc70), which recognizes KFERQ motifs and transports them to the lysosomal membrane [15]. Lysosomal-associated membrane protein 2A is a lysosomal receptor that regulates the translocation of CMA substrates into the lysosomal lumen via a process facilitated by the luminal resident form of Hsc70 [14,16]. Dysregulation of CMA has been associated with neurodegeneration, aging, and metabolic regulation [13,17,18]. Therefore, obtaining a better understanding of the mechanism regulating CMA could lead to the development of new therapeutic strategies for treating AD.

In this study, we aimed to investigate whether and how the compound ABG-001 produces anti-AD effects at lower concentrations via the gut–brain axis. We focused on identifying the target protein and mechanism of action of ABG-001 through experiments conducted on two different AD mouse models. Our findings demonstrated that ABG-001 improves the memory dysfunction in AD mice by activating CMA through IPA, which targets the Hsc70 protein and inhibits neuronal inflammation.

## 2. Results

### 2.1. ABG-001 Mitigates Cognitive and Behavioral Deficits in Memory in AD Model Mice

**Figure 1:**
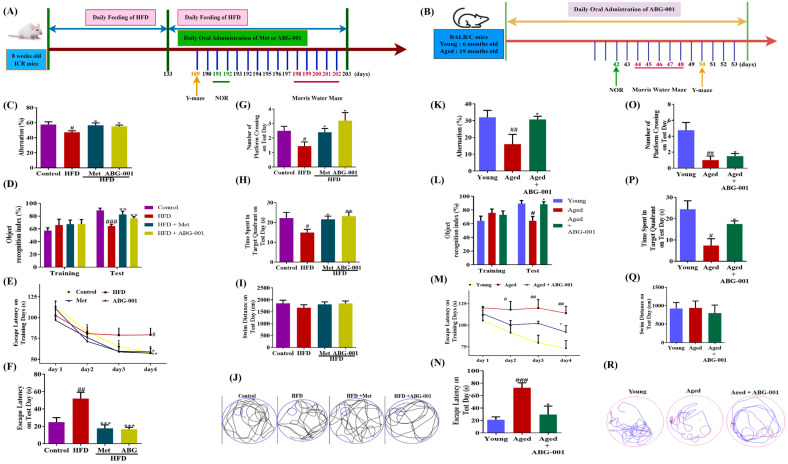
ABG-001 mitigates cognitive and behavioral deficits in memory of AD model mice. (**A**,**B**) Schedule of the HFD-induced AD mice experiment and the naturally aging mice experiment for the anti-AD effect of ABG-001. (**C**) Percentage alternation of HFD-induced AD mice in Y-maze test. (**D**) Object recognition index of HFD-induced AD mice in NOR test. (**E**) Escape latency of HFD-induced AD mice at the training phase in MWM test. (**F**) Escape latency in the test phase of MWM test in HFD-induced AD mice. (**G**) Changes on the number of platform crossings of HFD-induced AD mice. (**H**) Time spent on target quadrant. (**I**) Swim distance. (**J**) moving track of HFD-induced AD mice at the test phase. (**K**) Percentage alternation of naturally aging mice in Y-maze test. (**L**) Object recognition index of naturally aging mice in NOR test. (**M**) Escape latency of naturally aging mice in the training phase. (**N**) Escape latency of naturally aging mice on the test day of MWM test in naturally aging AD mice. (**O**) Number of platform crossings in naturally aging AD mice. (**P**) Time spent on target quadrant. (**Q**) Swim distance and (**R**) moving track of naturally aging AD mice at test phase. Big red circle, small red circle and blue line represented water maze, platform and moving track, respectively. # *p* < 0.05, ## *p* < 0.01, and ### *p* < 0.001 indicate statistical significance when HFD is compared with a normal control group or when aged mice are compared with young. * *p* < 0.05, ** *p* < 0.01, and *** *p* < 0.001 indicate statistical significance when HFD-only group is compared with drug-treated groups or when aged mice are compared with ABG-001 treated aged mice group.

In early research, we have investigated the dose-dependent effect of ABG-001 on HFD-induced AD mice at doses of 0.1, 5, 20 mg/kg. ABG-001 at a dose of 5 mg/kg exhibited the optimal effect on HFD-induced AD mice (Figure S2). These processes are illustrated in Figure 1A,B, respectively. Given that working and short-term memory impairment are recognized pathological features of AD, as is evident in both the naturally aged and HFD-induced AD mouse models [19,20], we employed the Y-maze and novel object recognition tests to evaluate the potential of ABG-001 to enhance these cognitive functions in AD mice. As shown in Figure 1C,D, the number of arm entries in each group was unaffected. However, the percentage of alternated mice in the HFD-only group was notably lower than that in the normal control group (Figure 1C, *p* < 0.05). Conversely, the metformin (Met) and ABG-001 treatment groups exhibited significantly greater percentages of alternations than the HFD-only group (Figure 1C, *p* < 0.05, *p* < 0.05). In the novel object recognition test, no significant differences in the object recognition or discrimination indices were observed during the training phase across all groups (Figure 1D). Nevertheless, lower recognition and discrimination indices were observed in the HFD-only group, while the Met and ABG-001 groups exhibited higher indices than did the HFD-only group (Figure 1D, *p* < 0.001, *p* < 0.01, *p* < 0.01). These results indicate that both ABG-001 and Met improve working and short-term memory in HFD-induced AD mice. Moreover, impaired spatial and long-term memory are prominent characteristics of cognitive decline in AD mouse models and patients and thus serve as crucial parameters for assessment [19,20]. Hence, we employed the MWM to evaluate these memory functions. During the four-day training phase, the escape latency in the HFD-only group was notably greater than that in the normal control group, while the escape latency was significantly shorter in the Met and ABG-001 groups than in the HFD-only group (Figure 1E). During the 5-day test phase, similar changes in escape latency were observed as during the training phase in the HFD-only group, Met group, and ABG-001 treatment group (Figure 1F, *p* < 0.01, *p* < 0.001, *p* < 0.001). Additionally, the number of platform crossings and the time spent in the target quadrant were significantly lower in the HFD-only group than in the control group (Figure 1G, *p* < 0.05; Figure 1H–J, *p* < 0.05). However, these parameters returned to normal levels following the administration of Met and ABG-001 (Figure 1G, *p* < 0.05, *p* < 0.05; Figure 1H–J, *p* < 0.05, *p* < 0.01). Furthermore, we conducted experiments using aging mice with AD to further validate the anti-AD effect of ABG-001, which yielded similar results to those observed in HFD-fed mice (Figure 1K, *p* < 0.01, *p* < 0.05, Figure 1L, *p* < 0.05, *p* < 0.05; Figure 1M, *p* < 0.01, *p* < 0.05; Figure 1N, *p* < 0.001, *p* < 0.05; Figure 1O, *p* < 0.01, *p* < 0.05; Figure 1P–R, *p* < 0.05, *p* < 0.05). These findings demonstrated that we successfully established an AD mouse model via HFD feeding and confirmed that ABG-001 at a dose of 5 mg/kg and Met at a dose of 140 mg/kg can ameliorate memory dysfunction in AD model mice. Importantly, the anti-AD effect of ABG-001 at a dosage of 5 mg/kg is comparable to that of Met at a dosage of 140 mg/kg.

### 2.2. ABG-001 Promotes Neuronal Protection and Neuro-Regeneration and Mitigates Synaptic Dysfunction in AD Mice

**Figure 2:**
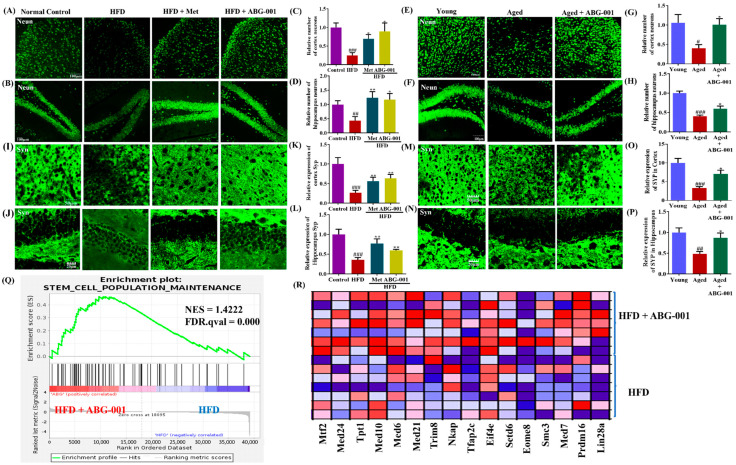
ABG-001 promotes neuronal regeneration and mitigates neuronal and synaptic dysfunction in AD model mice. (**A**,**B**) Photograph image of NeuN expression in the cortex and in the hippocampus of HFD-induced AD mice. (**C**,**D**) Digitalized results of NeuN expression in (**A**,**B**) with ImageJ. (**E**,**F**) Photograph of NeuN expression of cortex and hippocampus in naturally aging mice. (**G**,**H**) Digitalized results of NeuN expression in (**E**,**F**) with ImageJ v1.8.0. (**I**,**J**) Photograph image of Syp expression in the cortex and hippocampus of HFD-induced AD mice. (**K**,**L**) Digitalized results of Syp expression in (**I**,**J**). (**M**,**N**) Photograph image of Syp expression in the cortex and hippocampus of naturally aging mice. (**O**,**P**) Digitalized results of Syp expression in (**M**,**N**). Scale bars are 100 µm for the photograph of NeuN and 50 µm for the photograph of Syp. (**Q**,**R**) Gene set enrichment analysis for stem cell population maintenance and heat map. Red and blue colors represented positive and negative regulation, respectively. In the enrichment plot, the green line is the enrichment profile and the black vertical line is hits among the gene set. # *p* < 0.05, ## *p* < 0.01, and ### *p* < 0.001 indicate statistical significance when HFD is compared with a normal control group or when aged mice are compared with young. * *p* < 0.05, and ** *p* < 0.01 indicate statistical significance when an HFD-only group is compared with drug-treated groups or when aged mice are compared with an ABG-001-treated aged mice group.

To assess the impact of ABG-001 on the cognition of AD mice, a comprehensive analysis was conducted using immunohistochemical staining to investigate the changes in neuronal cells and synaptic function in the brain after treatment. Figure 2 visually demonstrates the notable decrease in neurons in the cerebral cortical and hippocampal regions in HFD-induced AD mice (Figure 2A,C, *p* < 0.001; Figure 2B,D, *p* < 0.01) and aging mice (Figure 2E,G, *p* < 0.05; Figure 2F,H, *p* < 0.001) compared with those in the normal control group. However, when HFD-induced AD mice and aging mice were treated with Met or ABG-001, a significant increase in the number of neurons in these areas was observed, surpassing the results of both the HFD-only group (Figure 2A,C, *p* < 0.05, *p* < 0.05; Figure 2B,D, *p* < 0.01, *p* < 0.05) and the young group (Figure 2E,G, *p* < 0.05; Figure 2F,H, *p* < 0.001). The corresponding synaptophysin (Syp) expression in the cortical and hippocampal regions is illustrated in Figure 2I–P. Notably, compared with those in the normal control group and young group, Syp expression in the cerebral cortex and hippocampus was significantly lower in the HFD-only group (Figure 2I,K, *p* < 0.001; Figure 2J,L, *p* < 0.001) and aging group (Figure 2M,O, *p* < 0.001; Figure 2N,P, *p* < 0.01), respectively. Conversely, the Met and ABG-001-treated groups exhibited noteworthy upregulation of these genes in the cerebral cortex and hippocampus of AD mice (Figure 2I,K, *p* < 0.01, *p* < 0.01; Figure 2J,L, *p* < 0.01, *p* < 0.01). Furthermore, similar changes in the cerebral cortex and hippocampus were also observed in aging mice after ABG-001 administration (Figure 2M,O, *p* < 0.05; Figure 2N,P, *p* < 0.05). These results indicate that ABG-001 enhances both the number of neurons and synapses in the cerebral cortex and hippocampus in AD model mice. Additionally, RNA sequence analysis was performed on cerebral cortex samples from HFD-induced AD mice. The results of the gene set enrichment analysis (GSEA) revealed that ABG-001 significantly increased the expression of genes associated with stem cell maintenance and differentiation (Figure 2Q,R, Table S1). Moreover, the expression of genes related to axon regeneration, neurogenesis (Table S1), and the assembly of tight junctions was increased. These findings imply that ABG-001 effectively upregulates the expression of genes crucial for neuronal regeneration. Overall, these results suggest that ABG-001 promotes neuronal function and regeneration and alleviates synaptic dysfunction in AD model mice.

### 2.3. ABG-001 Mitigates Glial Cell Activation and Neuroinflammation in Two AD Models Mice

**Figure 3:**
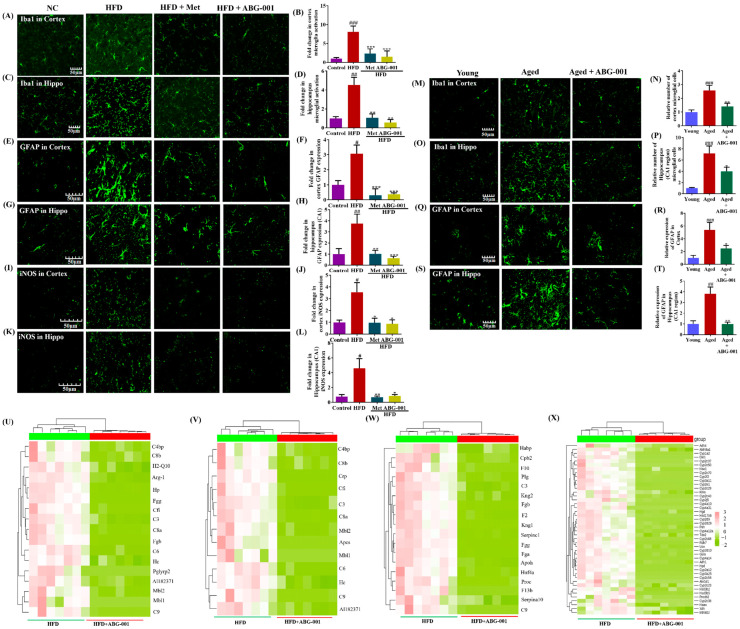
ABG-001 mitigates glial cell activation and neuroinflammation in AD model mice. (**A**–**D**) Photograph images and digitalized results of Iba-1 expression in the cortex and hippocampus CA1 region of HFD-induced AD mice. (**E**–**H**) Photograph image and digitalized results of GFAP expression in the cortex and hippocampus CA1 region of HFD-induced AD mice. (**I**–**L**) Photograph image and digitalized results of iNOS expression in the cortex and hippocampus CA1 region of HFD-induced AD mice. (**M**–**P**) Photograph image and digitalized results of Iba-1 expression in the cortex and hippocampus CA1 region in naturally aging AD mice. (**Q**–**T**) Photograph image and digitalized results of GFAP expression in the cortex and hippocampus CA1 region of naturally aging AD mice. (**U**–**X**) Changes in the gene expression-related immune system process, innate immune response, complement activation and classical pathway, and obsolete oxidation–reduction process in the cerebral cortex of AD mice after giving ABG-001. # *p* < 0.05, ## *p* < 0.01, and ### *p* < 0.001 indicate statistical significance when HFD is compared with a normal control group or when aged mice are compared with young. * *p* < 0.05, ** *p* < 0.01, and *** *p* < 0.001 indicate statistical significance when an HFD-only group is compared with drug-treated groups or when aged mice are compared with an ABG-001-treated aged mice group.

Since microglia, the resident cells of the brain, play vital roles in brain development, network maintenance, and injury repair [21,22,23], it is crucial to assess the effects of ABG-001 on microglial and astrocyte cells. Microglia function as CNS-regulated macrophages, eliminating threats such as microbes and protein aggregates and producing proinflammatory cytokines that influence neuronal inflammation and impact brain development, aging, and AD [21,22,23]. Astrocytes can also contribute to neuroinflammation, hyperphosphorylated Tau, and Aβ pathologies, leading to the formation of amyloid plaques and NFTs and neuronal dysfunction in AD [21,24]. To evaluate the effect of ABG-001 on microglial and astrocyte cells, we utilized biomarkers such as ionized calcium-binding adapter molecule 1 (Iba-1) and glial fibrillary acidic protein (GFAP). In mice fed HFD, compared with those in the normal control group, a significant increase in Iba-1 and GFAP expression was observed in the cerebral cortex and hippocampus (Figure 3A,B,E,F, *p* < 0.001, *p* < 0.05; Figure 3C,D,G,H, *p* < 0.01, *p* < 0.01). However, these parameters were effectively reduced in both the ABG-001 and Met treatment groups (Figure 3A,B,E,F, *p* < 0.001, *p* < 0.001; *p* < 0.01, *p* < 0.001; Figure 3C,D,G,H, *p* < 0.01, *p* < 0.01; *p* < 0.001, *p* < 0.01). In addition, microglial activation in the cortex and hippocampus was markedly greater in the aged group than in the young group (Figure 3M–T, *p* < 0.001, *p* < 0.001, *p* < 0.001, *p* < 0.01). Significantly lower microglial activation was observed in the ABG-001 group than in the aging group (Figure 3M–T, *p* < 0.01, *p* < 0.05, *p* < 0.05, *p* < 0.01). Notably, the activation of microglia and astrocytes in the DG and CA3 regions of the hippocampus was significantly greater in naturally aged mice than in control mice but was decreased in the CA1 region after ABG-001 administration (Figure 3O,P,S,T). These results indicate that ABG-001 mitigates the activation of glial cells in AD model mice.

The complement-dependent pathway involving glial cells plays a crucial role in pruning excess synapses during development. However, inappropriate activation of this pathway can lead to synapse loss in AD [25]. Furthermore, inflammatory agents such as interleukin-1 (IL-1), interferon-γ, and NF-κB can induce the expression of inducible nitric oxide synthase (iNOS) in astrocytes and microglia [26]. Therefore, we focused on measuring the expression of iNOS in the cortex and hippocampus. A marked increase in iNOS expression was observed in the HFD-only group compared with the normal control group (Figure 3I,J, *p* < 0.05; Figure 3K,L, *p* < 0.05). However, a significant decrease in iNOS expression in the cerebral cortex and hippocampus was observed in the Met and ABG-001 groups (Figure 3I,J, *p* < 0.05, *p* < 0.05; Figure 3K,L, *p* < 0.01, *p* < 0.05). By analyzing the biological processes associated with the genes, we found that ABG-001 affects genes related to immune system processes (Figure 3U–X), the innate immune response, complement activation, classical pathways, and oxidation–reduction processes. Consequently, there was reduced enrichment of genes related to cytokine response and biological oxidation (Figure 3U–X). In summary, these results demonstrated that ABG-001 mitigates neuroinflammation in AD mice.

### 2.4. ABG-001 Modulates the Gut Microbiota Diversity and Composition and Metabolite Levels in AD Model Mice

The gut–brain axis plays a significant role in neurodegenerative diseases, including AD [27]. The influence of the gut microbiota and their metabolites on AD pathology has been established [6,7]. Therefore, to investigate the involvement of the gut–brain axis in the anti-AD effect of ABG-001, we conducted 16S RNA sequence analysis of mouse fecal samples following ABG-001 administration, and the results are presented in Figure S3. Alpha diversity analysis revealed that the HFD-only group had a greater Chao1 index and a greater number of observed OTUs than did the control group. In contrast, the groups treated with Met or ABG-001 exhibited lower Chao1 and Simpson indices than did the HFD group (Figure S3A,B). Beta diversity analysis via Jaccard distance, Bray–Curtis dissimilarity, and unweighted UniFrac distances demonstrated that the HFD-only group was distinct from the control group, while the Met and ABG-001 groups were similar to the control group (Figure S3C,D). These results indicated that ABG-001, at a dose of 5 mg/kg, and Met, at a concentration of 140 mg/kg, reversed gut dysbiosis.

To further explore the effect of ABG-001 on the relative abundance of gut microbes, we analyzed the abundances of the bacteria at the phylum and genus levels (Figure S3E,F). Figure S3E shows the results of the phylum analysis, which revealed a significant increase in *Bacteroidota*, *Actinobacteria*, *Bacteroidetes*, and *Cyanobacteria*, as well as a reduction in *Verrucomicrobiota*, in the HFD-only group compared with the normal control group. However, these parameters returned to normal levels after treatment. Figure S3F shows the results of the genus analysis, which demonstrated a significant increase in *Lactococcus*, *Ligilactobacillus*, *Gemella*, *Porphyromonas*, and *Negativibacillus* in the ABG-001-treated group compared with the HFD-only group. Interestingly, compared with those in the HFD-only group, the abundances of *Akkermansia, Gemella, F082_unclassified, Porphyromonas*, *Veillonellaceae_unclassified, Peptostreptococcus, Prevotella, Catonella, Ligilactobacillus*, and *Rs-E47_termite_group_unclassified* were significantly greater. Notably, *Lactococcus*, *Ligilactobacillus*, and *Negativibacillus* are known to be SCFA-producing bacteria [28,29,30]. Consequently, we investigated SCFA metabolites, including IPA, tryptamine, butyrate, and VPA, in fecal, serum, and cerebral cortex samples, as they are closely related to AD.

**Figure 4:**
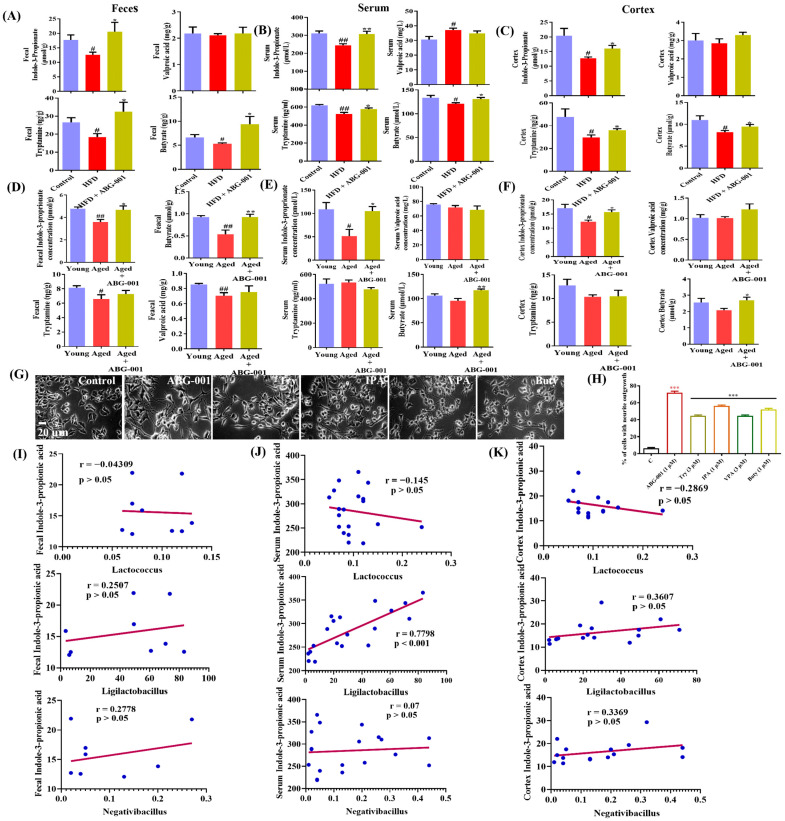
ABG-001 modulates gut microbiota composition and metabolites in AD model mice. (**A**–**F**) Effects of ABG-001 on gut metabolites, IPA, tryptamine, valproic acid, and butyrate in the feces, serum, and cerebral cortex of HFD-induced AD mice and naturally aging mice, respectively. (**G**) Micrographs of PC12 cells treated with ABG-001, Try, IPA, VPA and Buty. (**H**) Neurogenesis effects of these compounds in PC12 cells. (**I**–**K**) Correlation of IPA with *Lacotoccus*, *Ligilacillus*, and *Negativibacillus* in the feces, serum, and cerebral cortex of HFD-induced AD mice, respectively. Eight fecal, eight serums, and eight cerebral cortex samples from each group were used. # *p* < 0.05, and ## *p* < 0.01 indicate statistical significance when HFD is compared with the normal control group or when aged mice are compared with young. * *p* < 0.05, ** *p* < 0.01, and *** *p* < 0.001 indicate statistical significance when an HFD-only group is compared with drug-treated groups or when aged mice are compared with an ABG-001-treated aged mice group.

No change in the concentration of VPA in the fecal samples was observed in the HFD-only group compared with the normal control group. However, there was a significant reduction in IPA, tryptamine, and butyrate in the HFD-only group compared with those in the normal control group (Figure 4A, *p* < 0.05), and the levels returned to normal after ABG-001 treatment (Figure 4A, *p* < 0.05). In the serum samples, a significant reduction in IPA, tryptamine, and butyrate levels and a significant increase in VPA concentration were detected in the HFD-only group (Figure 4B, *p* < 0.01, *p* < 0.01, *p* < 0.05, *p* < 0.05) compared with the normal control group. These factors returned to normal levels after ABG-001 treatment (Figure 4B, *p* < 0.01, *p* < 0.05, *p* < 0.05). In the cerebral cortex, no change in VPA was observed in the HFD-only group compared with the normal control group. However, there was a significant reduction in IPA, tryptamine, and butyrate in the HFD-only group, and these factors significantly increased following ABG-001 treatment (Figure 4C, *p* < 0.05). These results were also observed in the samples from naturally aging mice (Figure 4D–F, *p* < 0.05, *p* < 0.01).

Furthermore, ABG-001 treatment led to a marked increase in the concentration of serum butyrate and IPA in aged-only mice compared with that in aged-only mice not treated with ABG-001. To investigate the neurodegenerative effect of these SCFAs, we evaluated their bioactivity in PC12 cells. Treatment with IPA, tryptamine, butyrate, or VPA resulted in a significant increase in the neuronal regeneration of the PC12 cells (Figure 4H). These results suggest that ABG-001 can regulate the gut microbiota to produce SCFAs, such as IPA, tryptamine, and butyrate, which can enter the blood and brain to promote neuritogenicity. Correlation analysis was also conducted to examine the interactions between the gut microbiome and gut metabolites. Figure S4A shows positive correlations between *Firmicutes* and serum IPA and tryptamine concentrations, as well as between cortex IPA and butyrate. In contrast, negative correlations were observed between *Bacteroidetes* and serum IPA/tryptamine and between *Bacteroidetes* and cortical IPA. *Proteobacteria* exhibited a negative correlation with the serum IPA. These results indicate that an increase in *Firmicutes* may contribute to higher levels of serum/cortex metabolites. Additionally, we conducted correlation analysis to identify the gut microbiota involved in IPA production, and the results suggested that *Ligilactobacillus* is a potential producer of IPA in the gut (Figure 4I–K). Correlation analysis also revealed positive correlations between serum IPA and object recognition/cortex NeuN expression, as well as negative correlations with escape latency (Figure S4B). Similar associations were observed with the serum butyrate/tryptamine ratio, cortical metabolite levels, and alternation/cortex NeuN expression, and these associations were negatively correlated with escape latency. These findings indicate that ABG-001 promotes the growth of beneficial SCFA-producing bacteria, increases the concentration of IPA and butyrate in the serum and brain, and facilitates neuron regeneration in AD model mice.

### 2.5. IPA Interacts with Hsc70 In Vitro and In Vivo

**Figure 5:**
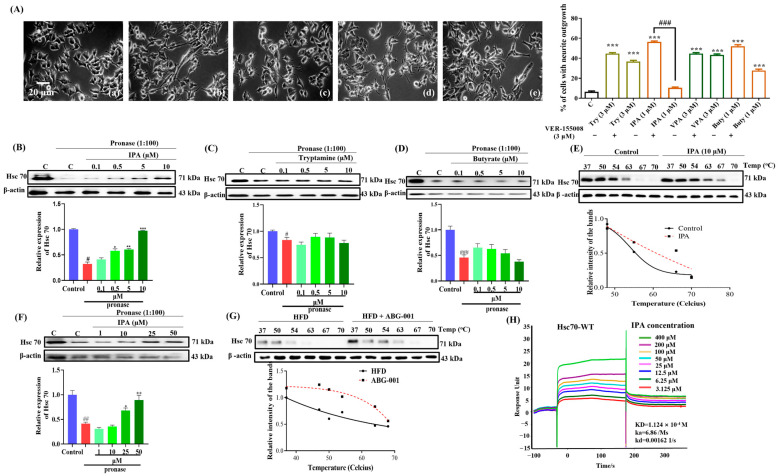
IPA interacts with Hsc70 in vitro and in vivo and target confirmation by CETSA and inhibitor analysis. (**A**) (**a**) The effect of an inhibitor of the Hsc70 protein on NGF mimics the effect of Try (**b**), IPA (**c**), VPA (**d**) Buty (**e**) in PC12 cells. The **b**, **c**, **d** and **e** represented Try, IPA, VPA and Buty. (**B**–**D**) DARTS and Western blot analysis and digital results of them for the Hsc70 protein with of IPA, tryptamine, and butyrate in PC12 cells. (**E**) CETSA and Western blot analysis and digital results of them for the Hsc70 protein with IPA in PC12 cells. (**F**,**G**) DARTS, CESTA, and Western blot analysis, and digital results of them for the Hsc70 protein with IPA in the cerebral cortex of AD mice. (**H**) The result of SPR analysis for the Hsc70 protein and IPA. #, ##, and ### indicate significant differences at *p* < 0.05, *p* < 0.01, and *p* < 0.001 when a pronase-only group is compared with a control group. *, **, and *** indicate significant differences at *p* < 0.05, *p* < 0.01, and *p* < 0.001 when a pronase-only group is compared with a pronase + drug group.

To identify the target protein of ABG-001, we employed the DARTS and CETSA techniques to screen potential candidates. Hsc70 inhibition revealed that Hsc70 inhibitor VER-155008 significantly reduced the neurogenesis of IPA in PC12 cells (Figure 5A). DARTS experiments revealed that tryptamine and butyrate had no effect on the stability of the Hsc70 protein, while IPA increased its stability in vitro (Figure 5B–E and Figure S5A). CETSA and DARTS were further utilized to confirm these findings in both cellular and animal models. Consistent changes in the thermal stability of the Hsc70 protein were observed, confirming the results of the DARTS analysis (Figure 5F,G and Figure S5B,C). To provide direct evidence of the interaction of IPA with Hsc70, we constructed an Hsc70 plasmid and purified Hsc70 protein and evaluated its affinity for IPA using SPR analysis. As anticipated, IPA exhibited strong affinity for the Hsc70 protein, with KD, ka, and kd values of 1.124 × 10^−4^ M, 6.86/Ms, and 0.00162 1/s (Figure 5H), respectively. These findings indicate that oral ABG-001 administration may enhance the production of IPA through modulation of the gut microbiota. In turn, IPA crosses the BBB and targets the Hsc70 protein in the brain, where it exerts its neurogenesis function.

### 2.6. IPA Serves as a Connecter of ABG-001 to Stabilize and Activate Hsc70 and Promotes the Degradation of CMA Substrates

**Figure 6:**
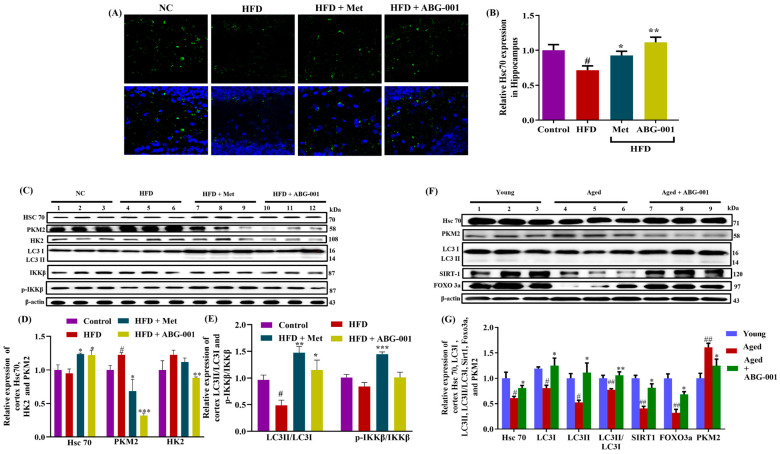
IPA as a connecter of ABG-001 activates Hsc70 and promotes the degradation of a CMA substrate. (**A**) Photograph image of Hsc70 expression in the hippocampus. (**B**) Hsc70 expression digitalized with Image J. (**C**) Western blot analysis of Hsc70, PKM2, HK2, LC3 I, LC3 II, IKKβ, p-IKKβ, and β-actin of the cortex of HFD-induced AD mice. (**D**) Represents digitalized Western blot results of Hsc70, PKM2, and HK2, respectively. (**E**) Represents digitalized Western blot analysis of the ratio of LC3 II to LC 3 I and the ratio of p-IKKβ and IKKβ in an HFD-induced AD mice model. (**F**) Western blot analysis of Hsc70, PKM2, LC3 I, LC3 II, SIRT1, FOXO3a, and β-actin of the cortex. (**G**) Represents digitalized Western blot result of Hsc70, LC3 I, LC3 II, LC3 II/LC3 I, SIRT1, and FOXO3a in naturally aged mice. The sample number of each group in Western blot is three, while the brains of three mice in each group were cut, and six sections and the hippocampus of each mouse were used to calculate the Hsc70 expression. Mean values ± SEMs are presented with statistical significance. # *p* < 0.05 and ## *p* < 0.01 indicate statistical significance when HFD is compared with the normal control group or when aged mice are compared with young. * *p* < 0.05, ** *p* < 0.01, and *** *p* < 0.001 indicate statistical significance when HFD only group is compared with drug-treated groups or when aged mice are compared with an ABG-001-treated aged mice group.

Hsc70 is a molecular chaperone that facilitates CMA, through which specific cytosolic proteins are targeted for degradation through the lysosome [14]. Our research showed that ABG-001 enhances the expression of IPA, which in turn targets the Hsc70 protein and promotes its stability. To investigate this further, we conducted immunohistochemical staining and Western blot analysis of the hippocampus and cerebral cortex of AD mice. We observed a significant decrease in Hsc70 expression only in the hippocampus of mice fed HFD compared with that in the control group (Figure 6A–D, *p* < 0.05; Figure S5A). However, in both the hippocampus and cortex, Hsc70 expression was significantly greater in the groups treated with Met or ABG-001 than in the HFD-only group (Figure 6A–D, *p* < 0.05, *p* < 0.01 for B; *p* < 0.05, *p* < 0.05 for D; Figure S6A). In addition, HK2 and PKM2 have been identified as substrates of CMA [31]. Thus, we examined whether the stabilization of Hsc70 promotes CMA by measuring the expression of key proteins in the downstream signaling pathway of CMA. We observed a significant increase in the expression of PKM2 and HK2 in the HFD-only group compared with the normal control group (Figure 6C,D, *p* < 0.05; Figure S6A). However, treatment with Met or ABG-001 significantly decreased the expression of PKM2 and HK2 (Figure 6C,D, *p* < 0.05, *p* < 0.001, *p* < 0.01; Figure S6A), respectively. Furthermore, we measured the expression of LC3 I and LC3 II and found that the ratio of relative LC3 II/LC3 I expression was significantly lower in the HFD-only group (Figure 6C,E, *p* < 0.05; Figure S6A). This ratio was significantly greater in the Met- and ABG-001-treated groups than in the HFD-only group (Figure 6C,E, *p* < 0.01, *p* < 0.05; Figure S6A). Considering that Met activates CMA by phosphorylating Hsc70 through p-IKKβ, we investigated whether ABG-001 also activates Hsc70 via a similar mechanism. We observed a significant increase in the p-IKKβ/IKKβ ratio in the Met group compared with that in the HFD-only group (Figure 6C–E, *p* < 0.001; Figure S6A). However, there was no significant difference in the p-IKKβ/IKKβ ratio in the ABG-001-treated group (Figure 6C–E and Figure S6A). These results suggest that the mechanism of action of ABG-001 differs significantly from that of Met. Additionally, we examined the effects of ABG-001 on protein alterations in the cerebral cortex of naturally aging mice. These mice exhibited changes in Hsc70, PKM2, LC3 I, and LC3 II levels and in the LC3 II/LC3 I ratio similar to those observed in HFD-induced AD mice (Figure 6F,G and Figure S6B, *p* < 0.05, *p* < 0.05, *p* < 0.05, *p* < 0.05, *p* < 0.01). In naturally aging mice, a reduction in SIRT1 and FOXO3a expression was noted in the aged group compared with the young group (Figure 6F,G and Figure S6B, *p* < 0.01, *p* < 0.01). However, the levels of these proteins in the cerebral cortex significantly increased after ABG-001 treatment in the aged group (Figure 6F,G and Figure S6B, *p* < 0.05, *p* < 0.05). Our findings demonstrated that ABG-001 enhances the production of IPA through gut microbiota regulation. IPA then crosses the BBB and targets the Hsc70 protein, facilitating the degradation of CMA substrates such as APP, phosphorylated Tau, and Aβ. Notably, the mechanism by which ABG-001 activates Hsc70 differs from that of Met.

### 2.7. As a Bridge for ABG-001, IPA Mitigates Aβ and Tau Pathology in the Cerebral Cortex and Hippocampus of AD Model Mice According to CMA

**Figure 7:**
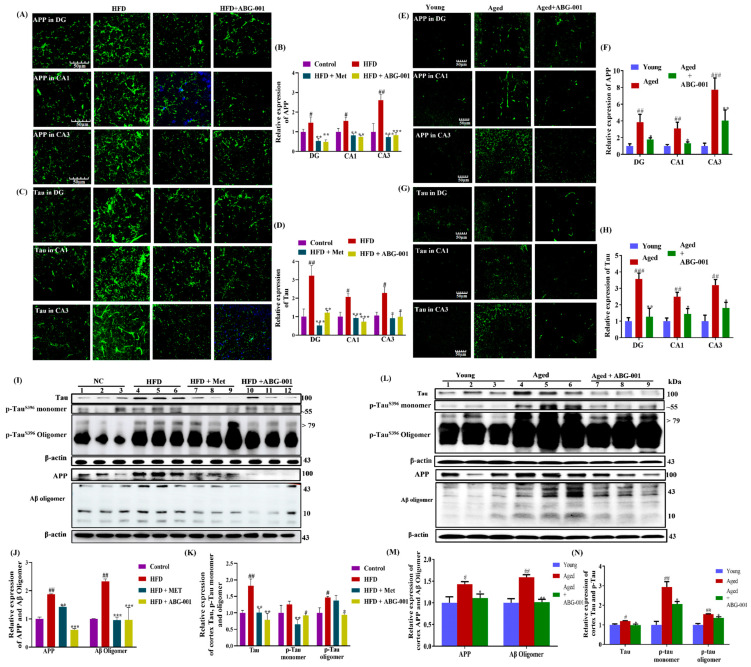
ABG-001 mitigates Aβ and Tau pathology in the cortex and hippocampus of AD model mice. (**A**,**B**) Photograph and digital results of APP expression in the DG, CA1, and CA3 regions of the hippocampus of HFD-induced AD mice. Blue color represented nucleus. (**C**,**D**) Photograph and digital results of Tau expression in the DG, CA1, and CA3 regions of the hippocampus of HFD-induced AD mice, respectively. (**E**–**H**) Photograph and digital results of Tau and APP expression in the DG, CA1, and CA3 regions of the hippocampus of naturally aging mice. (**I**–**K**) Western blot analysis of cortex Tau, p-Tau, APP and Aβ expression and digitalized result of Western blot analysis in HFD-induced AD mice. (**L**–**N**) Western blot analysis of cortex Tau, p-Tau, APP, and Aβ expression and digitalized result of Western blot analysis in naturally aging mice. The sample number for each group in the Western blot analysis is three, while the brains of three mice (HFD-induced AD mice) or two mice (naturally aging mice) in each group were cut, and six sections and the hippocampus of each mouse were used to calculate the protein expression. # *p* < 0.05, ## *p* < 0.01, and ### *p* < 0.001 indicate statistical significance when HFD is compared with the normal control group or when aged mice are compared with young. * *p* < 0.05, ** *p* < 0.01, and *** *p* < 0.001 indicate statistical significance when an HFD-only group is compared with drug-treated groups or when aged mice are compared with an ABG-001-treated aged mice group.

The deposition of Aβ and the hyperphosphorylation of the Tau protein in the brain are widely recognized as important biomarkers of AD [32]. Therefore, we conducted a study to measure the expression of APP, Aβ, Tau, and phosphorylated Tau (p-Tau) in the DG, CA1, and CA3 regions of the hippocampus, as well as in the cerebral cortex. Our findings revealed significant increases in APP and Tau expression in the DG, CA1, and CA3 regions in mice in the HFD group compared with those in the normal control group (Figure 7A,B, *p* < 0.05, *p* < 0.05, and *p* < 0.01; Figure 7C,D, *p* < 0.01, *p* < 0.05, and *p* < 0.05). However, after ABG-001 and Met were administered, APP and Tau expression in these regions was significantly lower than that in the HFD-only group (Figure 7A,B, *p* < 0.01, *p* < 0.01; *p* < 0.01, *p* < 0.01; *p* < 0.001, *p* < 0.001; Figure 7C,D, *p* < 0.01, *p* < 0.001; *p* < 0.001, *p* < 0.001; *p* < 0.05, *p* < 0.05). Similar findings were observed in aging mice. Additionally, we examined the expression of APP, Aβ oligomers, p-Tau monomers, and oligomers in the cortices of AD mice (Figure 7E,F, *p* < 0.01, *p* < 0.01, *p* < 0.001, *p* < 0.001; Figure 7G,H, *p* < 0.001, *p* < 0.01, *p* < 0.01). These parameters were also significantly increased in AD mice but decreased significantly after treatment with ABG-001 and Met (Figure 7I–K and Figure S7A,B, *p* < 0.01, *p* < 0.001; Figure 7L–N and Figure S7A,B, *p* < 0.05, *p* < 0.01). These results suggest that HFD and aging contribute to cognitive impairments through the regulation of APP, Aβ, Tau, and p-Tau protein expression in different regions of the hippocampus and cortex. Furthermore, ABG-001, which has Aβ and Tau-clearing properties, has potential as a therapeutic option for AD.

## 3. Discussion

ABG-001 is a derivative of gentisides from *Gentiana rigescens* Franch, a Chinese herbal medicine. It is a leading compound known for its neuroprotective, nerve regeneration, and antidiabetic effects [10,11,12,33]. To investigate the anti-AD effects of ABG-001 in different AD models, we employed HFD-induced AD mice, naturally aging mice with AD, and animal behavior experiments. The results in Figure 1 indicate that long-term HFD consumption leads to metabolic and memory dysfunctions, and the HFD-induced AD model has a high incidence and stable pathological state. ABG-001 at doses of 1 and 5 mg/kg effectively improved dysfunction in naturally aging mice and HFD-induced AD mice. Notably, the anti-AD effect of ABG-001 at a dose of 5 mg/kg was comparable to that of Met at a dose of 140 mg/kg. These findings are consistent with previous reports [34], highlighting the potential of this small molecule as a universal anti-AD agent.

The brain is a vital organ that controls cognitive ability, and the characteristic features of AD include the loss of neurons and a reduction in synapses. To gain a deeper understanding of the effect of ABG-001 administration on the brains of AD mice, we employed immunohistochemical staining and RNA-seq analysis to investigate the cerebral cortex and hippocampus. Figure 2 shows the changes in the number of neurons and synapses and the expression of genes related to neuron regeneration in the tissues of AD mice following the administration of ABG-001. These findings illustrate that long-term consumption of an HFD induces memory dysfunction in mice by impairing neurons and synapses in the cerebral cortex and hippocampus, whereas ABG-001 counters this pathological change in AD mice by facilitating neuron regeneration.

In addition to neuron inflammation being a pivotal factor in AD [22,34], glial cells, such as microglia and astrocytes, which influence cognitive abilities, produce proinflammatory cytokines. These inflammatory mediators play a crucial role in driving neuroinflammation and are associated with the hyperphosphorylation of Tau protein and Aβ pathologies, ultimately leading to the formation of amyloid plaques, NETs, and subsequent neuronal dysfunction observed during the aging process and in the cortex of AD patients [21,22,23,24]. Therefore, we examined biomarkers of inflammation, such as Iba1, GFAP, and iNOS, and analyzed the enrichment of genes related to the response to cytokines, biological oxidation, and complement activation. Figure 3 clearly shows the significant reductions in microglial and astrocyte activity, as well as iNOS activity, in the hippocampus and cerebral cortex and shows the changes in the expression of genes related to cytokines, biological oxidation, and complement activation after ABG-001 treatment. These findings confirmed that ABG-001 has an anti-AD effect by modulating neuronal inflammation.

Recently, there has been a significant focus on the relationship between the gut microbiota and human health. Multiple investigations have demonstrated the crucial role of the gut–brain axis in regulating various brain functions [35,36]. Moreover, metabolites produced by the gut microbiome can potentially promote nerve regeneration and protection [7,37]. Consequently, the gut microbiota have emerged as a promising target for pharmacological intervention aimed at mitigating age-related cognitive decline [6,7]. In this study, we also utilized 16S RNA sequence analysis technology to examine the gut microbiota. The results showed an increase in *Bacteroidetes* and a reduction in *Firmicutes* and *Ligilactobacillus* in the HFD group. Furthermore, there was a significant increase in the relative abundance of *Firmicutes* and *Ligilactobacillus* after the administration of ABG-001 (Figure S3E,F), indicating that ABG-001 had a substantial impact on the gut microbiota of AD mice. To provide additional direct evidence of the important role of the gut–brain axis in the neuroprotective effect of ABG-001, we measured SCFAs in the faces, serum, and cerebral cortex and NGF mimics the effects of them. The results presented in Figure 4 suggested that ABG-001 exhibited an anti-AD effect by increasing the levels of SCFAs, such as IPA, butyrate, VPA, and tryptamine, in the gut. Additionally, we found that *Lactobacillaceae* was positively correlated with IPA.

*Cyanobacteria* produce the neurotoxin β-N-methylamino-L-alanine (BMAA) to promote dysfunction within the nervous system and increase the formation of Aβ plaques [38]. Conversely, *Proteobacteria* can induce neuroinflammation and activate microglia, thereby accelerating cognitive decline [38,39]. *Staphylococcaceae*, specifically *Staphylococcus*, are known to produce LPS, which disrupts the BBB, leading to leaky brain activity and neuroinflammation [40,41]. In contrast, beneficial bacteria such as *Firmicutes*, which are found to be low in abundance in patients with AD, produce SCFAs that support cognitive function. *Lactobacillaceae* colonization in 5xFAD mice has been shown to reduce Aβ plaque deposition and advanced glycation end products RAGE [35]. Additionally, *Lactococcus* not only prevents Aβ plaque-induced paralysis and chemotaxis dysfunction in AD mice but also promotes increased lifespan [42]. The abundance of *Negativibacillus* was positively correlated with cognition [36]. During our study, the administration of ABG-001 significantly reduced the abundance of Gram-negative and pathogenic bacteria while increasing the abundance of beneficial Gram-positive bacteria and bacteria containing mobile elements (Figure S8). Gram-positive bacteria, specifically *Firmicutes*, produce short-chain fatty acids, including IPA, butyrate, VPA, and tryptamine. Furthermore, IPA and butyrate promote axonal regeneration and cognition and mitigate neuroinflammation [37,43,44]. VPA, an inhibitor of histone deacetylase, leads to the downregulation of cytokine expression [45]. As depicted in Figure 4, ABG-001 administration significantly elevated the concentrations of IPA, butyrate, and tryptamine in the serum, brain, and feces but did not increase the level of VPA. Notably, only IPA and butyrate were significantly increased in the serum, brain, and feces of both AD mouse models. A positive correlation was observed between beneficial gut microbiota and gut metabolites, as well as between gut metabolites and cognitive function markers (Figure S4A,B). These results indicate that the gut microbiota and their metabolites play a role in the anti-AD effect of ABG-001, with IPA potentially being the most important molecule in exerting the anti-AD effect of ABG-001. Hsc70, a crucial component of CMA, is strongly associated with AD [14]. In a previous study, we identified Hsc70 as a potential target of ABG-001 in vitro. However, during the pharmacokinetic study of ABG-001, we did not detect ABG-001 in the brain. Therefore, we propose that the effects of ABG-001 on gut metabolites may be instrumental in stabilizing and activating Hsc70 in vivo. To test this hypothesis, we employed DARTS and CETSA analyses to examine the influence of SCFAs from the gut on the affinity and stability of the Hsc70 protein. Moreover, we utilized SPR analysis to provide direct evidence that IPA specifically targets Hsc70. Our findings, as depicted in Figure 5, clearly demonstrate that only IPA can stabilize and activate Hsc70, making it the target protein of IPA. Given the crucial role of Hsc70 in CMA, a selective form of autophagy responsible for the lysosomal degradation of cytosolic proteins with KFERQ motifs [14], we further investigated the impact of IPA on PKM2, HK2, LC3 I, and LC3 II at the protein level. These proteins are key components of the autophagy pathway downstream of CMA. Significant decreases and increases in the expression of these proteins were observed in both the AD mouse group and the ABG-001-treated group, as illustrated in Figure 6, confirming the involvement of IPA, the ABG-001 linker, in Hsc70-mediated autophagy.

Alzheimer’s disease is characterized by two prominent pathological features: Aβ and Tau pathology. Specifically, the APP protein is responsible for generating Aβ peptides, which subsequently aggregate to form senile plaques in the brain [31]. Simultaneously, the Tau protein plays a vital role in stabilizing the microtubule network. When the Tau protein becomes hyperphosphorylated, it undergoes structural changes, leading to the formation of neurofibrillary tangles. The progressive buildup of Aβ and hyperphosphorylated Tau is closely associated with neuroinflammation, synaptic dysfunction, and cognitive impairment, all of which significantly contribute to the pathogenesis of AD [46,47]. To understand how IPA, as a connector of ABG-001, exerts its anti-AD effect in AD mice, we also examined the changes in Hsc70 subunits, including APP, Tau, and Aβ. As anticipated, the significant reduction in the expression of these proteins in the brains of ABG-001-treated AD mice, as illustrated in Figure 7, strongly supports our conclusion that IPA, the main metabolite of ABG-001, is involved in CMA and contributes to the clearance of Aβ and the phosphorylation of Tau in the hippocampus and cerebral cortex of AD mice.

Interestingly, Met can promote CMA by phosphorylating Hsc70 via p-IKKβ, as shown in a previous study [31]. However, the phosphorylation of IKKβ was not affected by ABG-001. These results suggested that Met and ABG-001 have unique mechanisms for activating Hsc70. Given the close relationship between aging and AD, we examined the expression of genes and proteins associated with aging and anti-aging biomarkers, such as SIRT1 and FOXO 3a, in naturally aging AD mice. ABG-001 significantly enriched genes related to FOXO 3a and SIRT1 and markedly increased the protein levels of FOXO 3a and SIRT1 (Figure 6F,G and Figure S6B). These findings demonstrated that ABG-001 improved memory dysfunction in AD mice by modifying aging signaling pathways.

In the present study, we found that ABG-001 regulates the gut microbiota to promote the production of IPA. IPA as a linker of ABG-001 targets the Hsc70 protein and regulates CMA signaling pathways. Regrettably, we did not obtain direct evidence that gut microbiota and IPA are involved in the anti-AD effect of ABG-001. Meanwhile, the binding sites of IPA and the Hsc70 or IGF-1R protein are also not clear. In the future, we will focus on these points to conduct research work.

## 4. Materials and Methods

### 4.1. Animals and Experimental Design

The anti-AD effect of ABG-001 on HFD-induced AD mice was evaluated in accordance with the data shown in Figure 1A. A total of 40 male Institute of Cancer Research (ICR) mice, aged 8 weeks, were obtained from the Zhejiang Academy of Medical Sciences in Hangzhou, China (animal use permit number SCXK (Zhejiang) 2019-0002). The mice were then housed under standard conditions with a 12 h light/dark cycle, a temperature of 23 °C, and 50% humidity. After 1 week of acclimatization, 10 mice were fed a normal chow diet (ND), while the remaining 30 mice were fed HFD. The HFD and ND were alternated every 2 days to maintain freshness. Weekly measurements were taken for water intake, food intake, and body weight. At the 18th and the 19th weeks, the animal behavior tests were conducted to evaluate the changes in memory of model mice. Once confirmation that an AD model was successfully established in the mice, characterized by impaired cognition (Figure S1), obesity, and hyperglycemia, the pharmacodynamic evaluation of ABG-001 against AD was conducted. Initially, the 10 mice in the ND group were assigned to the normal control group, while the remaining 30 mice in the HFD-induced AD group were divided into the HFD only group, the HFD + Met group, and the HFD + ABG-001 group. The normal control and HFD-only groups were orally administered an equal volume of water and soybean oil daily for 11 weeks. The AD mice in the Met and ABG-001 groups were administered at doses of 140 mg/kg and 5 mg/kg daily, respectively, over the same 11-week period. During the animal experiment, food intake, water intake, and body weight were measured weekly. Additionally, Y-maze, novel object recognition, and Morris water maze experiments were conducted during weeks 9, 10, and 11, respectively. The feces of mice in each group were obtained at the end of week 11. At the end of the animal experiment, blood was collected via the orbital sinus using a capillary tube, and the mice were euthanized by neck dislocation. The brain, liver, fat, pancreas, spleen, and intestine were then removed and preserved as samples for further analysis in a −80 °C freezer.

The anti-AD effects of ABG-001 on naturally aging AD mice were carried out: 12 female BALB/c mice (19 months old, 29–32 g) and 6 female C57BL/6j mice (8 weeks old, 29–32 g) were purchased from Biomice (Nantong, China, SCXK (Jiangsu) 2021-0003)). The mice were housed under standard conditions and acclimatized for 1 week. After that, the mice were divided into the young mouse group, aged mouse group, and aged mouse + ABG-001 group. The mice in the young group and aged group were orally administered soybean oil every day, while the aged + ABG-001 group received ABG-001 at a dose of 1 mg/kg dissolved in soybean oil. The experiment was conducted for 8 weeks, and the measurements of food intake, water intake, and body weight were taken. The NOR tests were performed in week 6, and the Morris water maze and Y maze tests were carried out in week 7. At the end of the experiment, the samples were collected as described in the above section. All animal experiments were conducted in accordance with the National Institutes of Health’s Guide for the Care and Use of Laboratory Animals and approved by the Zhejiang University Permission Committee on the Ethics of Animal Experiments (ZJU20230121, ZJU20230122).

#### 4.1.1. Y-Maze Test

The Y-maze test was used to assess working memory following a previously reported method [48]. The Y-maze had three arms (A, B, and C), each measuring 30 cm in length, 10 cm in width, and 15 cm in height, with a 120° angle relative to adjacent arms. Mouse activity was recorded using a video recorder, and the data were stored in ANY-MAZE software 6.35 (Stoelting, Chicago, IL, USA). In the test, each mouse was placed against the wall of arm A and allowed to freely move in the maze for 5 min. An arm entry was recorded when a mouse’s entire or half body entered an arm, and an alternation was considered when a mouse moved using all three arms without returning to the first arm. The total number of arm entries and alternations was calculated using ANY-MAZE software. Alternation was determined by dividing the total number of alternations by the total number of arm entries minus 2 and multiplying by 100.

#### 4.1.2. Novel Object Recognition (NOR) Test

The NOR test was conducted following a previously described protocol [48]. The test was performed in an open-field environment utilizing a black plastic box measuring 50 cm × 50 cm × 50 cm as the arena. The activity of the mice was recorded using a video recorder, and the footpad was saved in ANY-MAZE software. During the habituation phase, each mouse was allowed to explore the open arena for 5 min without any additional objects. Subsequently, the arena was cleaned with 75% ethanol and left to dry before proceeding with the training phase. The NOR test consisted of both a training phase and a testing phase, which took place 24 h after the habituation phase. In the training phase, each mouse was placed in the arena containing two identical steel objects, each measuring 5 cm × 5 cm × 5 cm and positioned equidistant from each other. The mice were given 5 min to explore these objects. After 1 h, one of the identical objects was replaced with a novel object, a cylindrical steel object measuring 5 cm × 5 cm, which was placed on top of each other. The time spent by each mouse exploring the objects was recorded during both the training and testing phases. Exploration included touching or sniffing the objects. These exploration durations were measured using a video recorder, and the resulting data were saved in ANY-MAZE software. ANY-MAZE software was used to calculate the object recognition index. This index was obtained by dividing the time spent exploring the novel object by the total time spent exploring both the novel and old objects and then multiplying by 100. The discrimination index was also calculated using ANY-MAZE software. This index involved subtracting the time spent exploring the novel object from the time spent exploring the old object and dividing the result by the sum of the time spent exploring both the novel and old objects.

#### 4.1.3. Morris Water Maze

The Morris water maze (MWM) test is a reliable method for evaluating the spatial and long-term memory abilities of mice. The maze apparatus consisted of a circular tank with a diameter of 1.25 m and a depth of 50 cm, filled with warm water (22 °C) to a depth of 25 cm. A submerged platform measuring 10 cm in diameter and located 1 cm below the water surface was utilized. Additionally, a video camera recorder and a computer were used to record and analyze the test sessions. The experiment consisted of 5 consecutive days, including a 4-day training period and a 1-day testing session. During the training phase, each mouse was placed at one of three different starting points in the maze, and the time needed for each mouse to locate the submerged platform was recorded. If a mouse failed to find the platform within 120 s, it was gently guided to the platform and allowed to remain there for 10 s. On the 5th day, the testing phase was carried out. The platform was removed, and the mouse was placed at a specific starting point. The mice were allowed to swim for 90 s in the maze. During this test session, the number of times the mouse crossed the platform, the time taken to escape, the time spent in the target quadrant, the average swimming speed, and the swimming distance were recorded.

### 4.2. Western Blot Analysis

Western blot analysis was conducted following a previously reported protocol [48]. The procedure involved homogenizing approximately 50 mg of cerebral cortex tissue and one hippocampus and collecting PC12 cells in SDS buffer containing 1% protease and 1% phosphatase inhibitors. The protein concentration was subsequently determined using a BCA kit. Subsequently, 10–30 µg of protein from each sample was transferred to individual tubes and denatured at 100 °C for 5 min. After denaturation, 30 µg of protein from each sample was loaded into separate wells of a sodium dodecyl sulfate–polyacrylamide gel. Gel electrophoresis was performed at 80 V for 15 min and then at 120 V for 60 min. The proteins within the gel were then transferred onto a polyvinylidene difluoride membrane. The membrane was subsequently blocked at room temperature for 60 min using a buffer solution containing 5% nonfat dry milk. The membrane was subsequently incubated overnight at 4 °C with primary antibodies against IKKβ, p-IKKβ, AMPK, p-AMPK, LC3B, Aβ, SIRT1, FOXO3a, (Cell Signaling Technology, Boston, MA, USA), APP, Tau, PKM2, HK2, (Proteintech, Wuhan, China), Hsc70, phospho-Tau S396 (Abcam, Boston, MA, USA), and β-actin (Beijing CoWin Biotech Company, Beijing, China). After three washes with Tris-buffered saline, the membrane was incubated with HRP-linked secondary antibodies (anti-mouse or anti-rabbit IgGs (Beijing CoWin Biotech Company, Beijing, China) for 45 min. The protein bands were then detected using an enhanced chemiluminescence (ECL) Western blot detection kit (Vazyme, Nanjing, China), and the blot density was quantified using ImageJ v1.8.0 software (National Institutes of Health, Bethesda, MD, USA).

### 4.3. Immunofluorescence Staining

Three mice from each HFD-induced AD group and two mice from the naturally aged AD group were anesthetized intraperitoneally. The tissues were then transcardially perfused with cold PBS, followed by cold 4% paraformaldehyde (PFA) in PBS to fix the brain tissue. The fixed brains were dehydrated in a 15% sucrose solution in PBS for 24 h and then transferred to a 30% sucrose solution for an additional 48 h. The brain was then excised and embedded in an optimal cutting temperature compound for sectioning. A cryostat (Thermo Fisher, Shanghai, China) was used to cut 20-µm-thick brain sections, which were placed in a well containing an anti-freeze agent (30% glycerin, 30% ethylene, and 40% PBS) and chilled to −30 °C. These brain sections were subjected to immunofluorescence staining. Initially, the brain sections were blocked by a blocking buffer for 1 h; the sections were washed three times with PBS, then incubated overnight at 4 °C with primary antibodies against proteins such as NeuN, Iba-1, GFAP, iNOS, Hsc70, phospho-Tau S396, and Aβ as well as Syp (Abcam, Cambridge, UK), Hsc70 (Abcam, Boston, MA, USA), APP, and Tau (Proteintech, Wuhan, China). The sections were washed three times with PBS and then incubated for 2 h at 37 °C with a 1:1000 dilution of the secondary antibody. Finally, the sections were covered with DAPI and a coverslip. Biomarker changes in brain tissue were examined using an upright two-photon confocal microscope (Olympus BX61, Tokyo, Japan). The neurons and the expression of GFAP, iNOS, APP, Tau, p-Tau, Aβ, and Syp in the hippocampus and cerebral cortex were quantified using ImageJ software (version 1.8.0).

### 4.4. Enzyme-Linked Immunosorbent Assay

The enzyme-linked immunosorbent assay (ELISA) was performed following established protocol [49]. At the conclusion of the animal experiments, blood samples were collected from the orbital sinus of each mouse using a capillary tube. These samples were allowed to sit at room temperature for 2 h before being centrifuged to separate the serum. The serum was then stored at −30 °C until further biochemical analysis. Additionally, cerebral cortex samples were homogenized with RIPA lysis buffer containing 1% protein protease inhibitor and 1% protein phosphatase inhibitors. Fecal metabolites were measured as previously described [49], with approximately 30 mg of fecal sample from each group being suspended in 1 mL of sterile pyrogen-free PBS in a tube. After gentle homogenization to prevent disruption of the bacterial cells, the mixture was centrifuged at 12,000× *g* for 15 min at 4 °C. The resulting supernatant was filtered using a 0.22 µm to 0.45 µm filter and then subjected to inactivation at 90 °C for 15 min. The supernatants from the control and treatment groups were diluted with PBS at specific ratios for the respective analytes. Commercially available mouse ELISA kits were utilized to determine the concentrations of these analytes in fecal samples, serum samples, and cerebral cortex samples. The optical density was ultimately measured at 450 nm using a microplate reader (BioTek, San Diego, CA, USA), and the concentrations of the analytes were calculated based on the standard curve for each analyte.

### 4.5. Cellular Thermal Shift Assay (CETSA)

A CETSA was performed as described in other reports [50]. First, approximately 2 × 10^6^ PC12 cells were separately added to four 60 mm dishes containing 5 mL of DMEM supplemented with 10% HS and 7.5% FBS (1% total) and incubated for 24 h. DMSO was added to two plates at a final concentration of 0.5% as a control group, and IPA was added to two plates at a final concentration of 10 μM as a treatment group. The plates were continuously incubated for 48 h. After that, the cells in the control group and treatment group were collected and divided into seven equal parts for the control or treatment, respectively. The seven equal cells of the control and treatment groups were heated at designated temperatures, such as 37, 50, 55, 60, 65 and 70 °C. Finally, Western blot analysis was used to detect changes in the Hsc70 protein. For the animal samples, cortical protein was extracted and heated at a temperature range of 50 °C to 70 °C, and Western blot analysis was used to detect the expression of the Hsc70 protein.

### 4.6. Drug Affinity-Responsive Target Stability (DARTS) Assay

The DARTS assay was conducted following established protocols [51]. For the cell culture experiments, 2 × 10^6^ cells were added individually to 60 mm dishes containing 5 mL of DMEM and incubated for 24 h. Subsequently, the proteins were extracted, and the protein concentration was measured. The proteins were then divided into groups of equal amounts and treated with IPA, tryptamine, or butyrate for 2 h in a shaker at concentrations of 0, 0.1, 0.5, 5, and 10 µM, respectively. Pronase was added to all the samples, except for the normal control, at a ratio of 1:100, and the samples were incubated for 25 min. For the animal samples, protein was extracted from the cerebral cortex, the protein concentration was measured, and 2 mg of protein was used for each group. The proteins were incubated with IPA at concentrations of 1, 10, 25, and 50 µM on ice overnight. Pronase was then added to all the groups, except for the normal control, at a ratio of 1:100 for 25 min. Subsequently, SDS-lysis buffer was added to each protein sample, and the samples were heated for 10 min before Western blot analysis was performed to quantify the expression of Hsc70.

### 4.7. Construction of the Hsc70 Expression Plasmid and Hsc70 Protein Purification

The cDNA of the mouse Hsc70 protein was cloned using RT–PCR and inserted into the pET-22b vector with a His tag via ligation. The resulting plasmid containing Hsc70 cDNA was transformed into Escherichia coli (BL21 (DE3)), and clones with the target gene were selected using agar plates containing ampicillin. The *E. coli* containing the recombinant plasmid was precultured overnight in 4 mL of autoclaved Luria–Bertani (LB) medium at 37 °C with shaking at 180 rpm. Then, 2 mL of the seed culture was inoculated into 1.5 L of LB medium and cultured at 37 °C with shaking at 180 rpm for 4 h until the logarithmic stage was reached. Next, 0.4 mM isopropyl thio-β-D-galactoside (IPTG) was added to initiate the expression of the target genes, after which the temperature was reduced to 18 °C. The cultures were incubated overnight, shaking at 150 rpm. The recombinant Hsc70 was then purified from intracellular proteins using sonication and Ni-affinity chromatography. After SDS–PAGE analysis and verification, the eluent was dialyzed and concentrated to obtain high-purity Hsc70 protein.

### 4.8. Surface Plasmon Resonance (SPR) Analysis

SPR analysis was conducted using a Biacore T200 instrument (GE Healthcare Life Sciences, Irvine, UT, USA). Initially, the purified His-tagged Hsc70 protein was immobilized onto an activated carboxymethylated 7 (CM7) chip (GE Healthcare Life Sciences, Irvine, UT, USA) using amine coupling, resulting in levels ranging from 12,000 RU to 14,000 RU. Next, IPA was dissolved in a PBS-P buffer solution (0.05% (*v*/*v*) Tween 20 and PBS) at a concentration of 400 μM. The IPA solution was then diluted to concentrations of 200, 100, 50, 25, 12.5, 6.25, and 3.125 μM using a PBS-P buffer solution. The sample was filtered through a 0.2 mm membrane. A gradient concentration of IPA was injected into the running buffer at a flow rate of 30 μL/min. The data were analyzed using Biacore evaluation software (T200 version 2.0) and fitted to the 1:1 Langmuir binding model to derive the kinetic parameters.

### 4.9. Gut Microbiota Analysis

At the conclusion of the animal experiment in the 11th week, fresh fecal samples were collected from each mouse and immediately stored at −80 °C for gut microbiota analysis. The analysis was conducted following a previously established protocol [49]. In summary, total genomic DNA was extracted from the samples using the TIANamp Bacteria DNA Kit (DP302-02, Tiangen, Beijing, China), and the V4 region of the 16S rRNA gene was amplified using a combination of forward and reverse primers. The broken sticky ends of the amplicon fragments were repaired using Klenow DNA polymerase, T4 DNA polymerase, and T4 PNK under PCR-specific conditions. The purified amplicons were pooled after magnetic bead purification and replicate PCR. Sequence analysis, including clustering sequences with ≥97% similarity and assigning operational taxonomic units (OTUs) to each representative sequence, was performed using UPARSE software (version 7.0, DCloud, Beijing, China). LC-Bio Technology Co., Ltd., in Hangzhou, China, conducted this analysis.

### 4.10. RNA Extraction Library Construction and Sequencing

Total RNA was extracted from cerebral cortex samples from mice using TRIzol reagent (Thermo Fisher, Waltham, MA, USA, 15596018) following the manufacturer’s procedure. The quantity and purity of the total RNA were subsequently analyzed using a Bioanalyzer 2100 and an RNA 6000 Nano LabChip Kit (Agilent, Santa Clara, CA, USA, 5067-1511). Only high-quality RNA samples with a RIN > 7.0 were selected for construction of the sequencing library. After the extraction of total RNA (5 µg), the mRNA was purified using Dynabeads Oligo (dT) (Thermo Fisher, Waltham, MA, USA) after two rounds of purification. The purified mRNA was then fragmented into short fragments using divalent cations at elevated temperature (Magnesium RNA Fragmentation Module (NEB, cat. e6150, Ipswich, MA, USA) under 94 °C 5–7 min). The fragmented RNA was reverse-transcribed using SuperScript™ II Reverse Transcriptase (Invitrogen, cat. 1896649, Carlsbad, CA, USA) to produce cDNA. cDNA was used to synthesize U-labelled second-strand DNAs with *E. coli* DNA polymerase I (NEB, cat.m0209, Ipswich, MA, USA), RNase H (NEB, cat.m0297, Ipswich, MA, USA), and dUTP Solution (Thermo Fisher, cat. R0133, Waltham, MA, USA). A blunt end was added to each strand, preparing them for ligation to the indexed adapters. The adapters, each containing a T-base overhang, were ligated to the A-tailed fragmented DNA. Dual-index adapters were subsequently ligated to the fragments, and size selection was performed with AMPure XP beads. Following the treatment of the U-labelled second-strand DNAs with the heat-labile UDG enzyme (NEB, cat.m0280, Ipswich, MA, USA), the ligated products were amplified via PCR. The PCR procedure included initial denaturation at 95 °C for 3 min; 8 cycles of denaturation at 98 °C for 15 s, annealing at 60 °C for 15 s, and extension at 72 °C for 30 s; and a final extension at 72 °C for 5 min. The average insert size for the final cDNA libraries was 300 ± 50 bp. Finally, the Illumina NovaSeq™ (Illumina, San Diego, CA, USA) 6000 system was used to perform 2 × 150 bp paired-end sequencing (PE150) following the vendor’s recommended protocol. This analysis was finished by LC-Bio Technology Co., Ltd., Hangzhou, China.

### 4.11. Biostatistics Analysis

Biostatistical analyses were conducted using GraphPad Prism 9.0 (GraphPad Software, San Diego, CA, USA) to perform one-way or two-way ANOVA, followed by a Dunnett *t*-test. For the analysis of gut microbiota data, either Wilcoxon or Kruskal tests were performed using R software (version 3.1.1). The results are presented as the mean ± standard error of the mean, and *p* < 0.05 indicates statistical significance.

## 5. Conclusions

**Figure 8:**
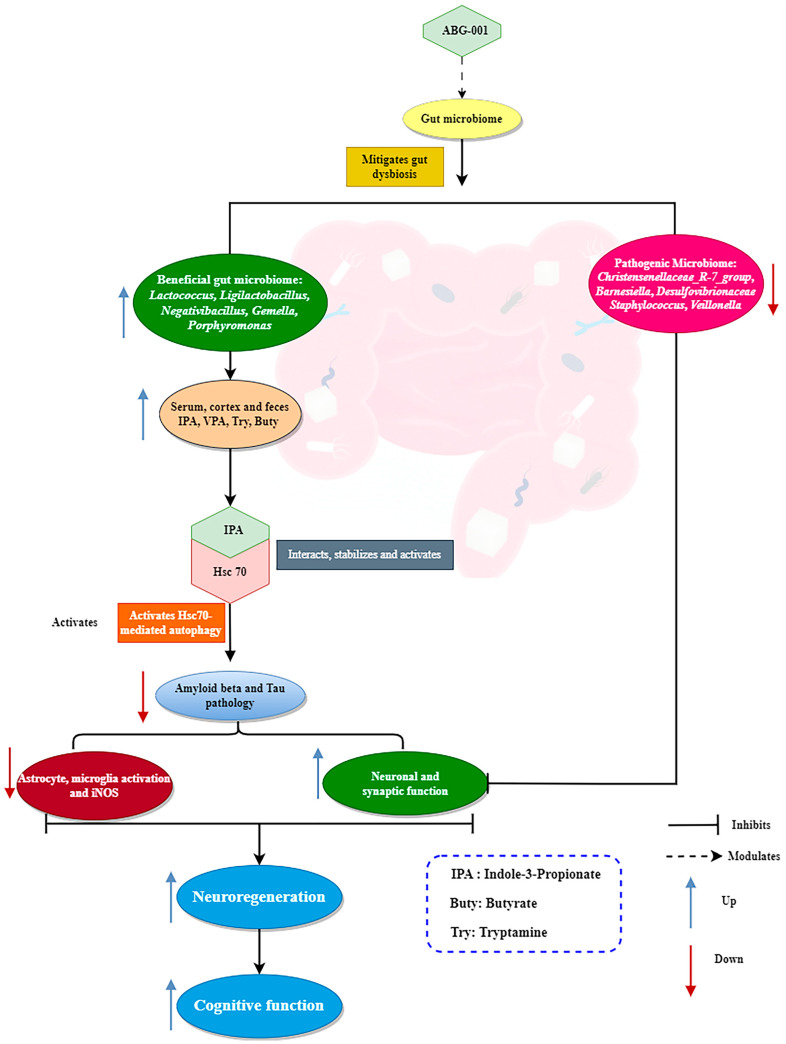
Proposed mechanism of action for ABG-001. ABG-001 activates the CMA pathway by IPA produced in the intestine to target the Hsc70 protein, and improves the memory dysfunction of AD mice.

In summary, this study investigated the therapeutic effect of ABG-001 on AD mice and revealed that ABG-001 alleviates memory dysfunction through neuron regeneration and protection. Additionally, ABG-001 promoted the growth of beneficial bacteria, such as *Lactococcus*, *Ligilactobacillus*, *Gemella*, *Porphyromonas*, and *Negativibacillus*, leading to increased levels of gut-derived metabolites, such as IPA. IPA enters the bloodstream, crosses the BBB, binds to and stabilizes Hsc70, and promotes CMA to clear Aβ and phosphorylated Tau proteins in the brain (Figure 8).

In the future, we will utilize fecal microbiota elimination and transplantation to obtain direct evidence on which gut microbiota and IPA are involved in the beneficial effect of ABG-001. Meanwhile, the protein crystallization and cryo-electron microscopy techniques will be used to determine the binding sites of IPA and Hsc70 or IGF-1R protein, respectively.

## Data Availability

All figures and data used to support this study are included within this article.

## Supplementary Materials

The following supporting information can be downloaded at https://www.mdpi.com/article/10.3390/ijms252111719/s1.
