# Supplementary material for: Tetradecyl 2,3-Dihydroxybenzoate Improves Cognitive Function in AD Mice by Modulating Autophagy and Inflammation Through IPA and Hsc70 Targeting
Source: Int J Mol Sci. 2024 Oct 31;25(21):11719. doi: 10.3390/ijms252111719 (PMC11547019; doi:10.3390/ijms252111719)
Supplement: Supplementary file 1 [file ijms-25-11719-s001.zip › ijms-3282305-supplementary.pdf]

Supplementary Information

Supplementary Table S1. The RNA sequence analysis for the cerebral cortex samples of HFD-induced AD mice experiment.

Name	Size	ES	NES	NOM. P val	FDR. qval
HEMATOPOIETIC_STEM_CELL_DIFFERENTIATION	15	0.5491341	1.5141860	0.040618956	8.773056e-01
STEM_CELL_POPULATION_MAINTENANCE	64	0.4642010	1.4221518	0.089397090	1.000000e+00
REGULATION_OF_NEUROGENESIS	37	0.4655328	1.2915206	0.149402400	1.000000e+00
STEM_CELL_DIFFERENTIATION	37	0.4615962	1.2608883	0.189134800	1.000000e+00
NEURONAL_STEM_CELL_POPULATION_MAINTENANCE	24	0.4241373	1.1792941	0.271484380	1.000000e+00
SOMATIC_STEM_CELL_POPULATION_MAINTENANCE	39	0.4043669	1.0972974	0.356997970	1.000000e+00
AXON_REGENERATION	17	0.4160279	1.0893999	0.359315600	1.000000e+00
NEUROGENESIS	63	0.3111682	1.0454416	0.398797600	1.000000e+00
SPINAL_CORD_DEVELOPMENT	35	0.3384760	0.9438246	0.547619040	1.000000e+00
SPINAL_CORD_MOTOR_NEURON_DIFFERENTIATION	23	0.2877925	0.9343643	0.586956500	1.000000e+00
NEURON_DEVELOPMENT	61	0.2546867	0.8849301	0.663306500	1.000000e+00
MYELINATION	54	0.3192982	0.8704274	0.662857100	1.000000e+00
CEREBRAL_CORTEX_DEVELOPMENT	78	0.2471993	0.8612326	0.696428600	1.000000e+00
POSTSYNAPTIC_ACTIN_CYTOSKELETON_ORGANIZATION	18	0.3158074	0.8544190	0.622047250	1.000000e+00
PERIPHERAL_NERVOUS_SYSTEM_DEVELOPMENT	22	0.3148607	0.8456158	0.702213300	1.000000e+00
REGULATION_OF_NEURON_DIFFERENTIATION	32	0.2486282	0.7514588	0.840996150	1.000000e+00

Supplementary Figures

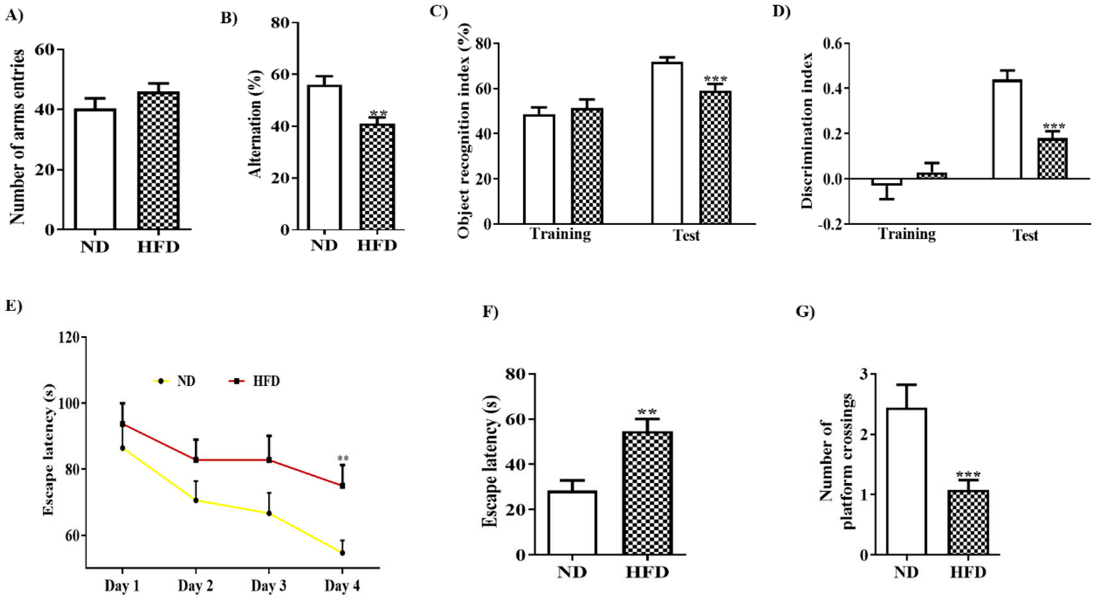

Supplementary Figure S1. The analysis results of animal behavior experiments for normal mice and HFD mice.

(A)-(B) the numbers of entranced arms and percentage alternation of normal mice and HFD mice in Y-maze test. **(C)-(D)** Object recognition index and discrimination index of normal mice and HFD mice in NOR test. **(E)** Escape latency of normal mice and HFD mice at the training phase in MWM test. **(F)-(G)**Escape latency and changes on the number of platform crossings in the test phase of MWM test in normal mice and HFD mice.

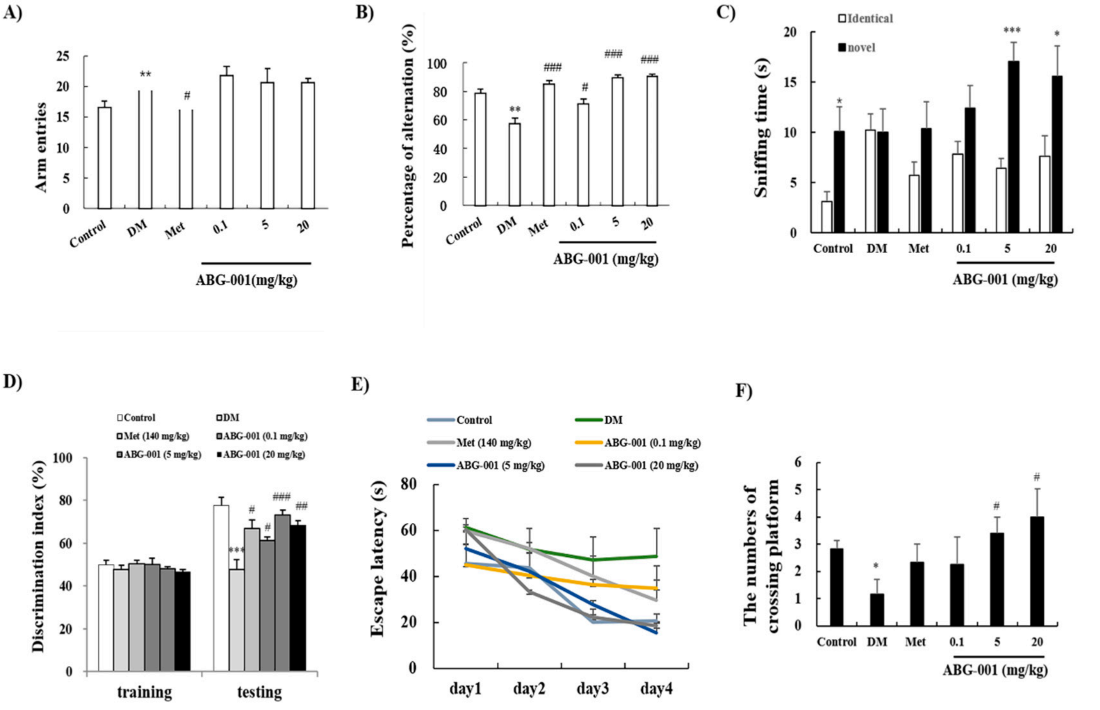

Supplementary Figure S2. The effect of ABG-001 on HFD-induced AD mice at doses of 0.1, 5 and 20 mg/kg. A)-B) The results of Y maze test. C-D) The results of novel object recognition test. E-F) The results of water maze test. ND and fifty HFD cognitive impaired mice into six groups: control (ND-fed mice), negative control group (untreated HFD-fed mice), positive control (MET 140 mg/kg plus HFD diet) and ABG-001 groups (ABG-001 plus HFD diet). ABG-001 treatment groups included 0.1, 0.5 and 20 mg/kg dose groups. Animal numbers of each group are ten. $^{*}p<0.05$, $^{**}p<0.01$ and *** $p<0.001$ represent significant different compared with normal control. $^{\#}$ $p<0.05$, $^{\#\#}p<0.01$ and $^{\#\#\#}p<0.001$ indicate significant different compared with pathological model group (DM).

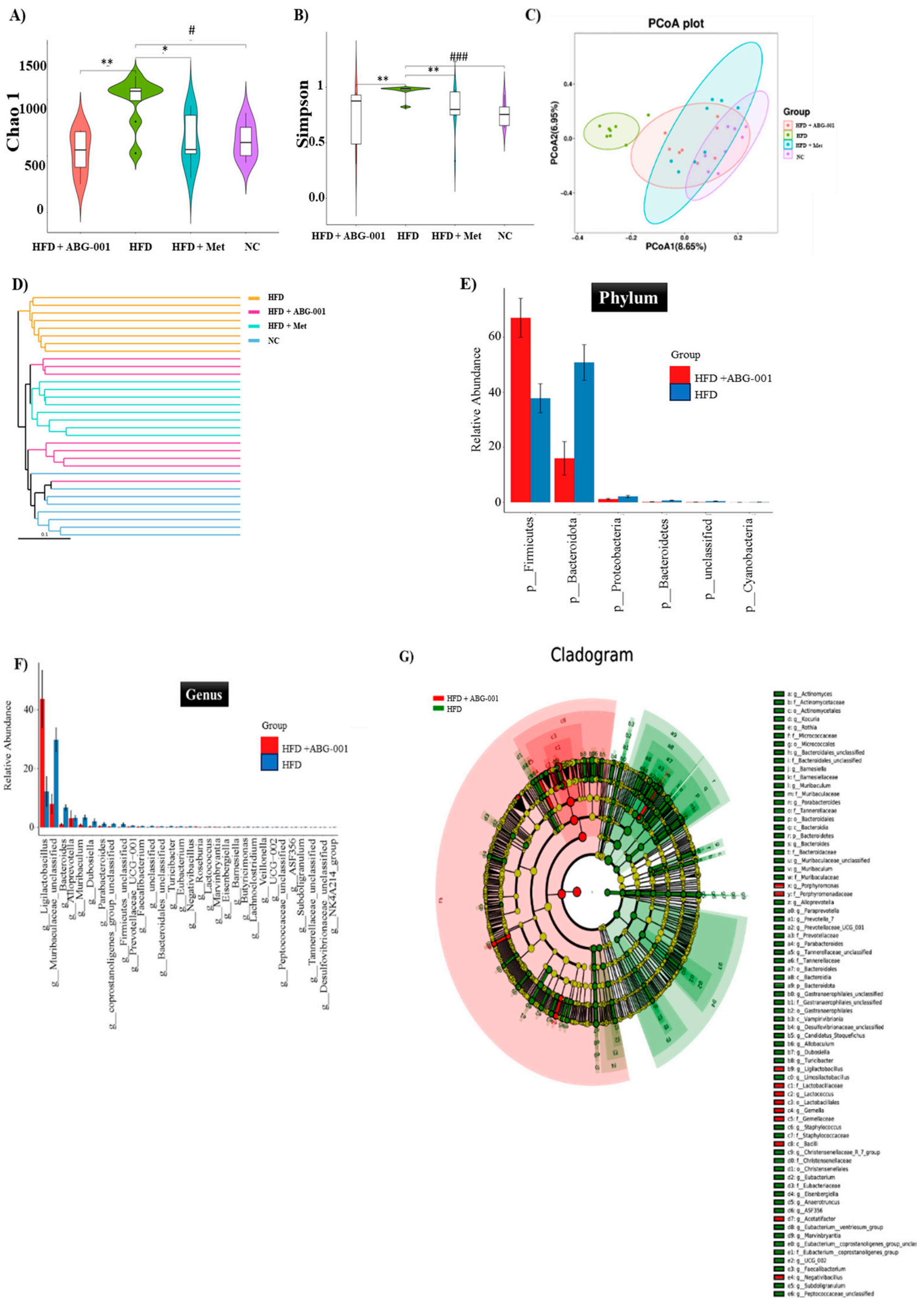

Supplementary Figure S3. Effect of ABG-001 on the gut microbiota of HFD-induced AD mice.

(A) The results of alpha diversity analysis for gut microbiota of HFD-induced AD mice in Chao. **(B)** Sampson levels. **(C)** The results of Principal coordinate analysis. **(D)** cluster analysis in bata diversity analysis. The changes on relative abundance of gut microbiota at phylum **(E)** and genus **(F)** levels after administrating ABG-001. **(G)** The cladogram map of gut microbiota

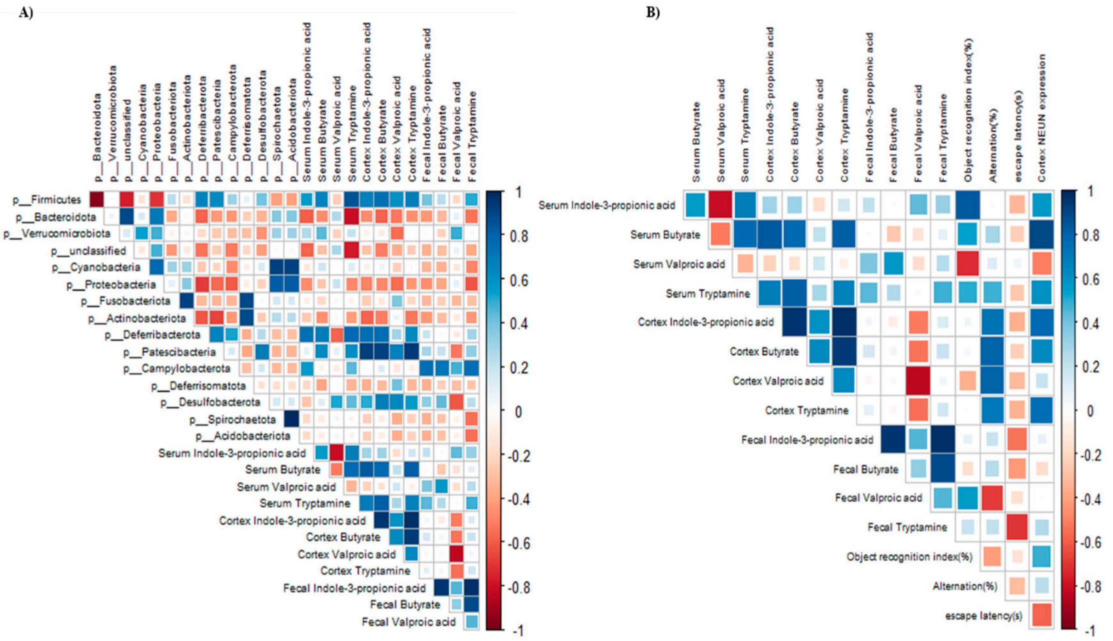

Supplementary Figure S4. (A) The correlation between gut microbiota and IPA, valproic acid and butyrate in fecal, serum and cerebral cortex. **(B)** The correlation among IPA, valproic acid and butyrate in fecal, serum and cerebral cortex, and object recognition, alternation, escape latency.

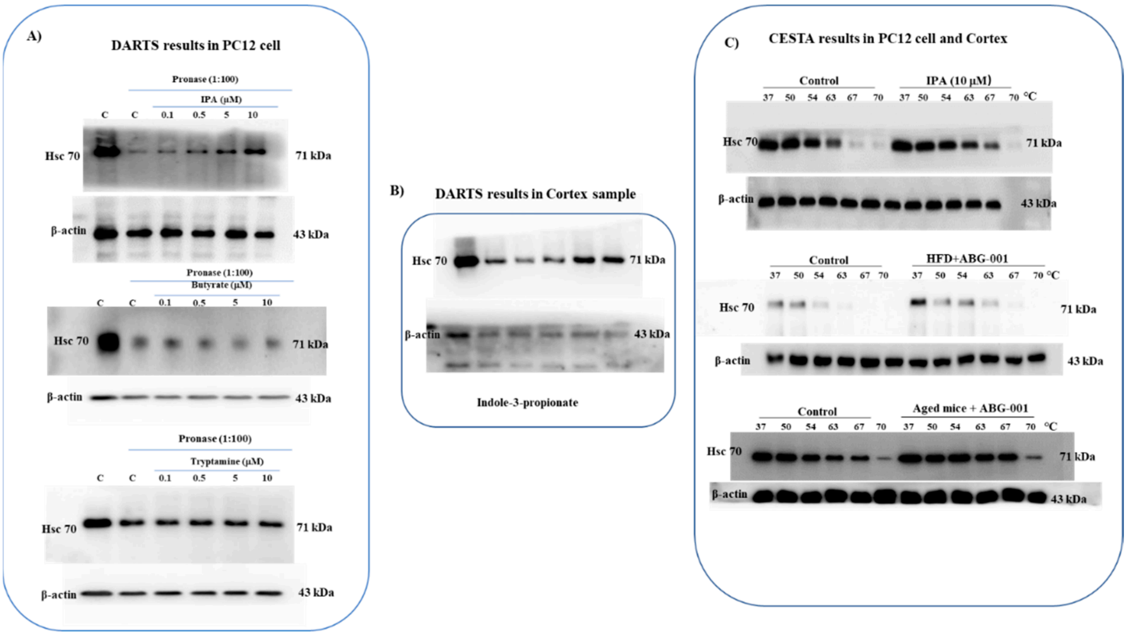

Supplementary Figure S5. The origin data of western blot analysis for DARTS experiment of IPA, Butyrate, tryptamine at PC12 cells in Figure **(A)** and **(B)** DARTS experiment of IPA with cerebral cortex. **(C)** The western blot analysis for CETSA experiment in PC12 cells of IPA, cerebral cortex samples of HFD-induced AD mice and nature aging mice after giving ABG-001.

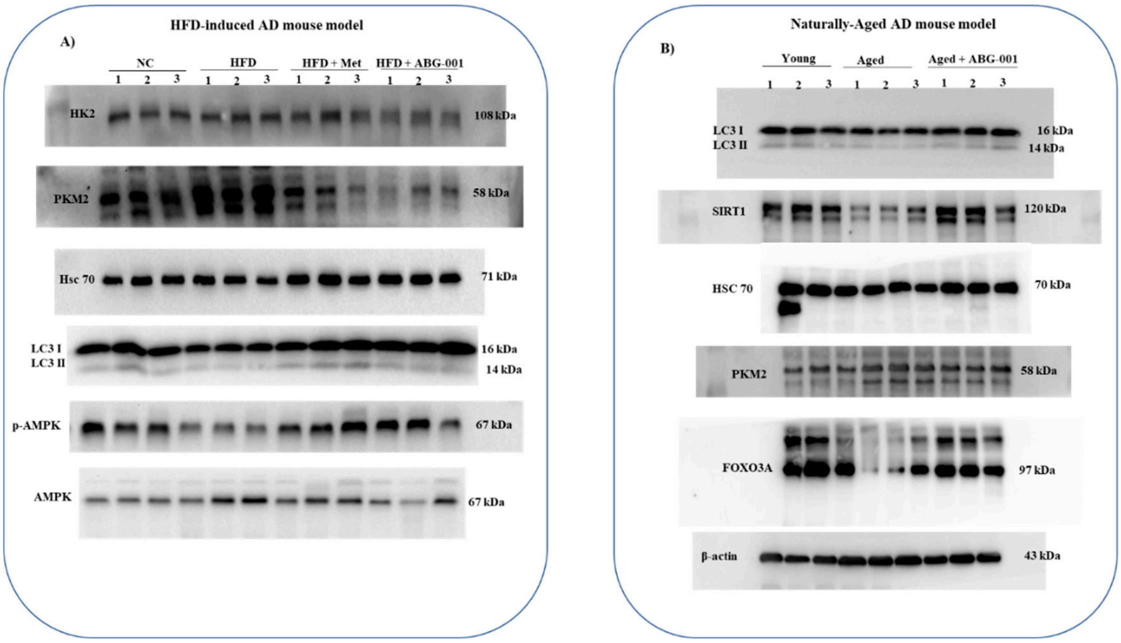

Supplementary Figure S6. (A) The origin data of western blot analysis for HM2, PKM2, Hsc70, LC3B, p-AMPK, AMPK in HFD-induced AD mice after treating ABG-001. **(B)** The origin data of western blot analysis for LC3B, Hsc70, SIRT1, FOXO3A, PKM2 in nature aged AD mice after treating ABG-001.

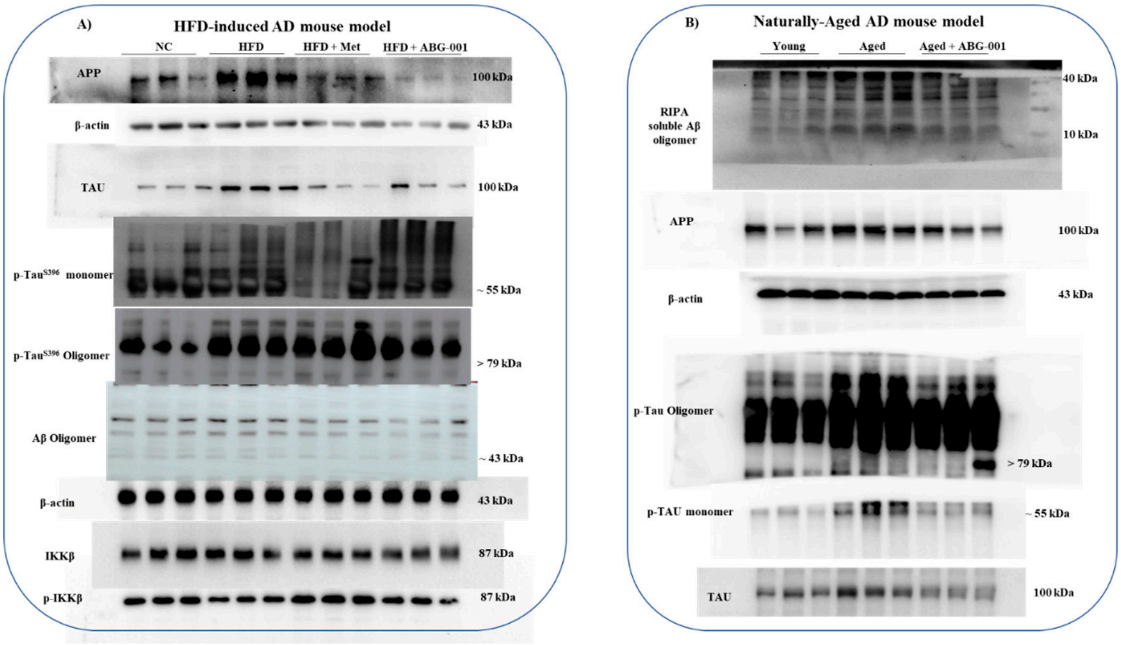

Supplementary Figure S7. (A) The origin data of western blot analysis for APP, Tau, phosphorylated-Tau, IKKβ and phosphorylated-IKKβ in HFD-induced AD mice after treating ABG-001. **(B)** The origin data of western blot analysis for Ab, APP, Tau, and phosphorylated-Tau in naturally aging AD mice after treating ABG-001.

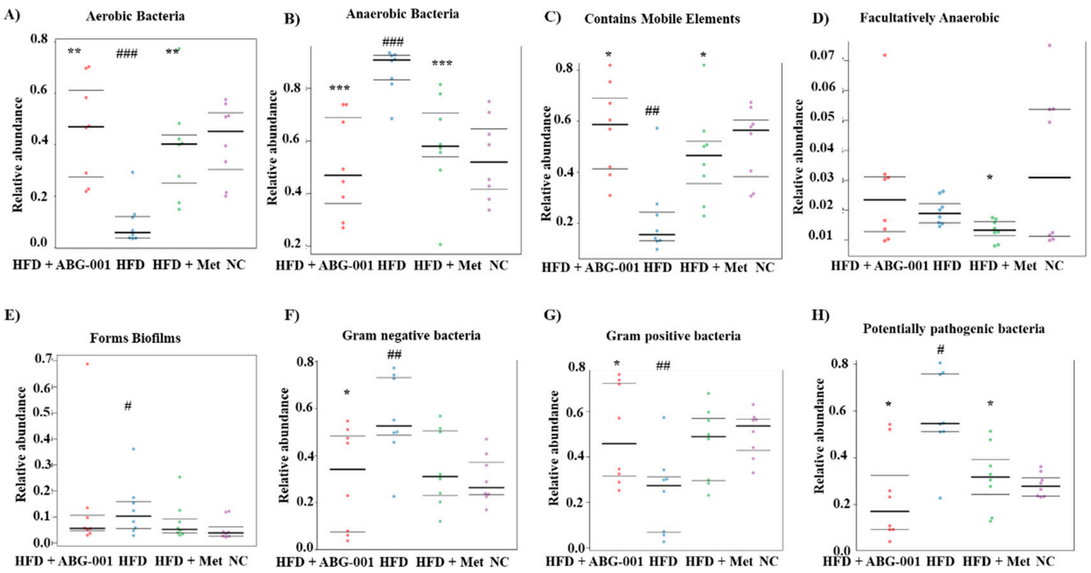

Supplementary Figure S8. Effect of ABG-001 and Metformin on gut microbiota phenotypic predictions. Graphical representation of gut microbiota predicted phenotypes such as **(A)** Aerobic, **(B)** Anaerobic, **(C)** Contains mobile elements, **(D)** Facultatively Anaerobic, **(E)** Forms Biofilms, **(F)** Gram Negative, **(G)** Gram Positive, **(H)** Potentially Pathogenic in all experimental groups of AD mice.
